# Metal ions govern coronavirus endoribonuclease activity

**DOI:** 10.1093/nar/gkaf1508

**Published:** 2026-01-14

**Authors:** Xionglue Wang, Jing Li, Zhichao Liu, Longfei Wang, Bin Zhu

**Affiliations:** Key Laboratory of Molecular Biophysics, the Ministry of Education, College of Life Science and Technology, Huazhong University of Science and Technology, Wuhan430074, China; Department of Cardiology, Taikang Center for Life and Medical Sciences, Zhongnan Hospital of Wuhan University, School of Pharmaceutical Sciences, Wuhan University, Wuhan430072, China; Key Laboratory of Molecular Biophysics, the Ministry of Education, College of Life Science and Technology, Huazhong University of Science and Technology, Wuhan430074, China; Department of Cardiology, Taikang Center for Life and Medical Sciences, Zhongnan Hospital of Wuhan University, School of Pharmaceutical Sciences, Wuhan University, Wuhan430072, China; Key Laboratory of Molecular Biophysics, the Ministry of Education, College of Life Science and Technology, Huazhong University of Science and Technology, Wuhan430074, China; Shenzhen Huazhong University of Science and Technology Research Institute, Shenzhen518063, China

## Abstract

Coronavirus nonstructural protein 15 (nsp15) is an endoribonuclease that restricts viral double-stranded RNA (dsRNA) accumulation in the cytosol to evade host immunity. Given the co-localization of nsp15 and replicating viral RNA, the mechanism controlling nsp15 activity is essential, yet poorly understood. Although metal ions are widely used as cofactors for enzymes, their role in nsp15 remains elusive. Here, we show that Co^2+^ or Ni^2+^ potently activates, whereas Zn^2+^ inhibits nsp15 of multiple coronaviruses. In the presence of Co^2+^, cryo-electron microscopy structures of severe acute respiratory syndrome coronavirus 2 (SARS-CoV-2) nsp15/dsRNA complexes indicate higher dsRNA-binding affinity. Active-site mutation H249A weakens the effects of Co^2+^, Ni^2+^, and Zn^2+^ on SARS-CoV-2 nsp15. Furthermore, the Co^2+^- or Ni^2+^-dependent activation of nsp15 is inhibited upon Zn^2+^ addition, suggesting competitive regulation. Overall, our work identifies the activator and inhibitor ions of nsp15 and suggests a metal-dependent regulatory mechanism of nsp15 activity.

## Graphical Abstract

**Graphical Abstract:**
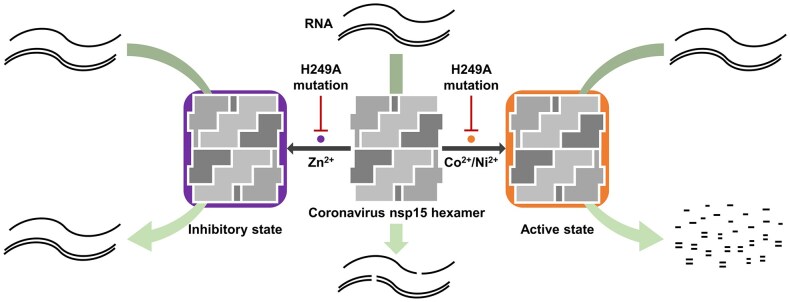

## Introduction

Interferon (IFN) responses are vital for vertebrate hosts to combat viral infections [1]. However, coronaviruses, especially highly pathogenic human coronaviruses including severe acute respiratory syndrome coronavirus (SARS-CoV), Middle East respiratory syndrome coronavirus (MERS-CoV), and SARS-CoV-2, can evade host IFN responses via multiple strategies [2, 3], promoting viral replication and pathogenesis [4–6]. The replication of the coronaviral positive-stranded RNA genome inevitably produces double-stranded RNA (dsRNA) as an intermediate [7], which is capable of triggering IFN responses through the activation of dsRNA sensors [8]. It is widely accepted that coronavirus nonstructural protein 15 (nsp15) facilitates evasion of dsRNA sensors by restricting viral dsRNA accumulation in the cytosol through its uracil-specific endoribonuclease (EndoU) activity [9–13], and several potential mechanisms based on cleavage of viral RNA have been proposed [14–16]. Significantly, given its co-localization with the viral replication and transcription complex (RTC) and replicating viral RNA [9, 17–20], nsp15 activity must be controlled in an ordered manner to ensure that viral RNA is not degraded by nsp15 during genome replication and transcription, and that residual viral dsRNA is degraded by nsp15 efficiently following the completion of viral RNA synthesis. However, the regulatory mechanism of nsp15 activity is poorly understood.

Although metal ions are widely used as cofactors for enzymes, their role in nsp15 remains elusive. Mn^2+^ was found to enhance the *in vitro* RNA cleavage activity of nsp15 [16, 21–25]. However, this enhancement was reported to be conditional and substrate-dependent [26, 27], and was usually observed at millimolar concentrations of Mn^2+^, a level that exceeds the physiological range [28, 29]. In addition, the effect of Mn^2+^ on nsp15 stability and oligomerization appears to be subtle [16, 26, 30–32], and all attempts to identify the presence of Mn^2+^ in nsp15 structure have been unsuccessful [24, 33, 34]. In this study, we discovered that both Co^2+^ and Ni^2+^ activate nsp15 significantly more potently than Mn^2+^. On the other hand, Zn^2+^ was found to have a pronounced inhibitory effect on nsp15 activity. We determined cryo-electron microscopy (cryo-EM) structures of SARS-CoV-2 nsp15/dsRNA complexes in the presence of Co^2+^ and identified that the active-site residue H249 is important for Co^2+^, Ni^2+^, and Zn^2+^ to exert their effects on SARS-CoV-2 nsp15. These findings suggest a metal ion-mediated regulatory mechanism of nsp15 activity.

## Materials and methods

### Protein expression and purification

#### For wild-type SARS-CoV-2 nsp15 and its mutants

The recombinant proteins with an N-terminal 6 × His tag of wild-type SARS-CoV-2 nsp15 (YP_009725310.1) and its H234A mutant (referred to as His-nsp15 and His-H234A, respectively) were expressed and purified with a two-step purification process as previously described [16]. The complete sequence of the His-nsp15 construct is listed in Supplementary Table S1.

To obtain the Strep-nsp15 and Strep-H234A constructs, the sequence coding Strep-Tag II (Trp-Ser-His-Pro-Gln-Phe-Glu-Lys) was introduced in place of the 6 × His tag sequence in the His-nsp15 and His-H234A constructs using the Gibson assembly method (primers for cloning are listed in Supplementary Table S2). To express Strep-nsp15 and Strep-H234A, the constructs were transformed into *E. coli* BL21(DE3)pLysS cells (ToloBio, China). These cells were cultured in 1 L (for Strep-H234A) or 4 L (for Strep-nsp15) of LB medium containing 50 µg/mL kanamycin and 34 µg/mL chloramphenicol at 37°C until an OD600 of approximately 0.8 was reached. The flasks containing the culture were then placed at 4°C for 1 h. Subsequently, protein expression was induced by the addition of 0.2 mM IPTG, and incubation continued at 16°C for 16 h. The cells were harvested and then stored at −80°C. To purify Strep-nsp15 and Strep-H234A, the frozen cells were thawed on ice and resuspended in a Strep binding buffer [100 mM Tris–HCl (pH 8.0 at 25°C), 300 mM NaCl, and 1 mM EDTA], then lysed by ultrasonication. The supernatant was collected after centrifugation at 23  710 × *g* and 4°C for 1 h and filtered with 0.45-μm filters. The filtered supernatant was loaded onto a StrepTrap XT 1 mL column (Cytiva, USA) pre-equilibrated with 10 volumes of the Strep binding buffer. The column was washed with 50 volumes of the Strep binding buffer. Most of the Strep-tagged nsp15 was eluted by a Strep binding buffer that had been supplemented with 40 mM biotin. The collected eluates were concentrated with an Amicon Ultra-15 30K centrifugal filter (Millipore, USA) and dialyzed against a storage buffer containing 50 mM Tris–HCl (pH 8.0 at 25°C), 100 mM NaCl, 0.1% (v/v) Triton X-100, and 50% (v/v) glycerol, and then stored at −20°C.

Amino acid mutations (H249A, K289A, T340A, and Y342A) were introduced in the His-nsp15 construct using the Gibson assembly method (primers for cloning are listed in Supplementary Table S2). These mutants were expressed with the same procedure as detailed above and purified using an Ni-NTA agarose column (Qiagen, Germany). Specifically, the frozen cells were thawed on ice and resuspended in a lysis buffer [50 mM Tris–HCl (pH 8.0 at 25°C), 500 mM NaCl, 5% (v/v) glycerol, and 10 mM imidazole], then lysed by ultrasonication. The supernatant was collected after centrifugation at 23  710 × *g* and 4°C for 1 h and filtered with 0.45-μm filters. The filtered supernatant was loaded onto the Ni-NTA agarose column pre-equilibrated with five volumes of the lysis buffer. The column was first washed with 10 volumes of an elution buffer [20 mM Tris–HCl (pH 8.0 at 25°C), 500 mM NaCl, 5% (v/v) glycerol, and the indicated concentration of imidazole] containing 30 mM imidazole, and then five volumes of an elution buffer containing 50 mM, 60 mM, 70 mM, 80 mM, and 200 mM imidazole. The eluates with 70 mM, 80 mM, and 200 mM imidazole were collected and mixed, then concentrated and dialyzed as detailed above.

#### For the nsp15 of other coronaviruses

The codon-optimized DNA fragments encoding nsp15 of SARS-CoV (NP_828872.1), MERS-CoV (YP_009047226.1), MHV-A59 (YP_009915685.1), PEDV (NP_839968.1), and HCoV-229E (NP_835355.1) were synthesized and inserted into pET-28a(+) vectors harboring an N-terminal 6 × His tag by GenScript. SARS-CoV nsp15, MERS-CoV nsp15, MHV-A59 nsp15, and HCoV-229E nsp15 were expressed with the same procedure as detailed above, with the exception of the use of Rosetta 2(DE3)pLysS cells (TOLOBIO, China), and were purified using the Ni-NTA agarose column as detailed above, except that only eluates with 80 mM and 200 mM imidazole were collected. The expression and purification process of PEDV nsp15 is somewhat different. First, the protein expression of PEDV nsp15 was induced by the addition of 1 mM IPTG, and incubation continued at 25°C for 16 h. In addition, a second purification step was performed. Specifically, the eluates with 70 mM, 80 mM, and 200 mM imidazole were collected, followed by their mixing and concentration. Subsequently, the concentrated sample was loaded onto a Superdex 200 Increase 10/300 GL column (Cytiva, USA) pre-equilibrated with a gel filtration buffer [20 mM Tris–HCl (pH 8.0 at 25°C), 500 mM NaCl, and 5% (v/v) glycerol] for gel filtration chromatography.

### Protein analysis

Protein concentration was determined with a Bradford Protein Quantitative kit (Bio-Rad, USA), with bovine serum albumin as a standard. Protein purity was analyzed by 12.5% SDS-PAGE with Coomassie blue staining. Protein size was determined with Precision Plus Protein Standards, 10 to 250 kDa (Bio-Rad, USA), PageRuler Plus Prestained Protein Ladder, 10 to 250 kDa (Thermo Scientific, USA), and 250 kDa Plus Prestained Protein Marker (Vazyme, China) in SDS-PAGE analysis.

### RNA substrates preparation

The SARS-CoV-2 mini-genome RNA was prepared as previously described [16]. The RNA oligonucleotides, including FRET-RNA, ssRNA15, ssRNA44, and the complementary strand of ssRNA44, were synthesized chemically by GenScript. dsRNA44 was generated through the process of annealing between ssRNA44 and its complementary strand. The RNA annealing reaction containing 10 mM Tris–HCl (pH 7.4 at 25°C), 50 mM KCl, 25 μM ssRNA44, and 25 μM the complementary strand of ssRNA44, was carried out on a PCR instrument with a specific annealing program (1. 90°C, 1 min; 2. 90°C, 5 s, −0.1°C per cycle; 3. GOTO step 2, 650×; 4. 4°C, ∞.).

### RNA cleavage assays

#### SARS-CoV-2 mini-genome RNA as substrates

600 ng of SARS-CoV-2 mini-genome RNA was incubated with 2 nM nsp15 to a final volume of 10 μL in an RNA cleavage buffer [50 mM Tris–HCl (pH 7.4 at 25°C), 140 mM KCl, zero or 1 mM DTT, and no divalent cations or 0.5 mM MnCl_2_, CoCl_2_, or NiCl_2_]. The reaction was performed at 37°C for 30 min and then terminated by the addition of 10 μL of 2 × RNA loading dye (New England Biolabs, USA). The samples were heated at 85°C for 2 min, immediately placed on ice for 2 min, and then analyzed by 1% TAE agarose gel electrophoresis with ethidium bromide staining. RNA was visualized with a UVsolo Touch system (Analytik Jena, Germany). Image processing was conducted using ImageJ software.

#### ssRNA44 or dsRNA44 as substrates

500 nM ssRNA44 or dsRNA44 was incubated with the indicated concentration of nsp15 or 5 pM RNase A (Thermo Scientific, USA) to a final volume of 10 μL in an RNA cleavage buffer [50 mM Tris–HCl (pH 7.4 at 25°C), 140 mM KCl, and no divalent cations or the indicated concentrations of MnCl_2_, CoCl_2_, NiCl_2_, ZnCl_2_, and CuCl_2_]. The reaction was performed at 37°C for 30 min and then terminated by the addition of 10 μL of 2 × RNA loading dye. The samples were heated at 85°C for 2 min, and immediately placed on ice for 2 min. To generate an RNA size ladder, alkaline hydrolysis of ssRNA44 at a concentration of 6 μM to a final volume of 10 μL in an alkaline hydrolysis buffer [50 mM sodium carbonate (pH 9.4) and 1 mM EDTA] was performed at 90°C for 15 min and quenched with 10 μL of 2 × RNA loading dye. All samples were analyzed by 20% TBE-urea PAGE (8 M urea). RNA was visualized with a ChemiScope imager (CLiNX, China) using the Cy2 (Ex470BL, Em525/30F) channel. Images were processed, and the grey values of gel bands were quantified with ImageJ software.

#### FRET-RNA as substrates

500 nM FRET-RNA was incubated with the indicated concentration of nsp15 to a final volume of 20 μL in an RNA cleavage buffer [50 mM Tris–HCl (pH 7.4 at 25°C), 140 mM KCl, and 5 mM EDTA, MnCl_2_, CoCl_2_, or NiCl_2_] at 37°C. The cleavage of the FRET-RNA substrate would result in an increase in fluorescence. Time-course reactions were directly monitored in a CFX Connect qPCR system (Bio-Rad, USA) using the FAM channel. The initial velocity was estimated using the slope from linear regressions of the reaction monitored during 1–5 min.

#### ssRNA15 as substrates

500 nM ssRNA15 was incubated with the indicated concentration of nsp15 to a final volume of 10 μL in an RNA cleavage buffer [50 mM Tris–HCl (pH 7.4 at 25°C), 140 mM KCl, and no divalent cations or the indicated concentration of MnCl_2_, CoCl_2_, NiCl_2_, ZnCl_2_, or CuCl_2_] (containing no DTT unless otherwise indicated). The reaction was performed at 37°C for 15 min and then terminated by the addition of 10 μL of 2 × RNA loading dye. The samples were heated at 85°C for 2 min, immediately placed on ice for 2 min, and then analyzed by 20% TBE-urea PAGE (8 M urea). RNA was visualized with a ChemiScope imager using the Cy2 (Ex470BL, Em525/30F) channel. Images were processed and the grey values of gel bands were quantified with ImageJ software.

### Detection of the hydrolysis of 2′,3′-cyclic phosphodiesters

500 nM ssRNA15 was incubated with 300 pM RNase A in an RNA cleavage buffer [50 mM Tris–HCl (pH 7.4 at 25°C) and 140 mM KCl] at 37°C for 15 min to produce 2′,3′-cyclic phosphodiesters. For the reaction with Mn^2+^, Co^2+^, or Ni^2+^, 1 μL of 50 mM MnCl_2_, CoCl_2_, or NiCl_2_ (1 μL of H_2_O here for the NoD control) and 1 μL of nsp15 solution were added to 8 μL of the RNase A reaction sample; for the reaction with Zn^2+^ or Cu^2+^, 5 μL of the mixture containing 50 mM Tris–HCl (pH 7.4 at 25°C), 140 mM KCl, 0.5 mM ZnCl_2_ or CuCl_2_, and 1000 nM nsp15 (the mixture contains no divalent cations for the NoD control) was added to 5 μL of the RNase A reaction sample. The reaction was incubated at 37°C for 15 min, followed by the additions of 1.25 μL of 10 × rCutSmart buffer (New England Biolabs, USA) and 1.25 μL of Quick CIP (New England Biolabs, USA). The reaction with CIP was performed at 37°C for 30 min and then terminated by the addition of 12.5 μL of 2 × RNA loading dye. The samples were heated at 85°C for 2 min, immediately placed on ice for 2 min, and then analyzed by 20% TBE-urea PAGE (8 M urea). RNA was visualized with a ChemiScope imager using the Cy2 (Ex470BL, Em525/30F) channel. Image processing was conducted using ImageJ software.

### EMSA

500 nM ssRNA44 or dsRNA44 was incubated with 7.8 μM His-H234A to a final volume of 10 μL in an RNA-binding buffer [50 mM Tris–HCl (pH 7.4 at 25°C), 140 mM KCl, and no divalent cations or 5 mM MnCl_2_, CoCl_2_, NiCl_2_, or ZnCl_2_] for 20 min at 37°C. Then, 2 μl of 6 × EMSA loading buffer [10 mM Tris–HCl (pH 8.0 at 25°C) and 60% (v/v) glycerol] was added into the mixture. The samples were analyzed by 12% TBE PAGE on ice. RNA was visualized with a ChemiScope imager using the Cy2 (Ex470BL, Em525/30F) channel. Image processing was conducted using ImageJ software.

### RNA binding assay

1 μM dsRNA44 was incubated with 5 μM His-H234A to a final volume of 1 mL in an RNA-binding buffer [50 mM Tris–HCl (pH 8.0 at 25°C), 100 mM NaCl, and zero or 0.5 mM MnCl_2_ or CoCl_2_] at 37°C for 10 min. Subsequently, the sample was loaded onto a Superdex 200 Increase 10/300 GL column pre-equilibrated with the RNA-binding buffer for gel filtration chromatography. The collected samples were further analyzed by 12.5% SDS-PAGE with Coomassie blue staining and 20% TBE PAGE with ethidium bromide staining. RNA was visualized with a UVsolo Touch system. Image processing was conducted using ImageJ software.

### Native-PAGE

10 μL of sample containing 50 mM Tris–HCl (pH 7.4 at 25°C), 140 mM KCl, zero or 0.5 mM CuCl_2_, and 6 μM nsp15 was incubated at 37 °C for 10 min. Then, 1 μL of 50 mM EDTA was added into the sample, followed by 10 min incubation at 37°C. After that, 3.7 μL of 4 × Native-PAGE loading buffer (Solarbio, China) was added to the mixture. The samples were analyzed by 7.5% Native-PAGE with Coomassie blue staining. Protein size was determined with High Molecular Weight Native Electrophoresis Protein Marker II (Real-Times, China). In addition, the samples were also analyzed by 12.5% SDS-PAGE.

### DSF analysis

25 μL of sample containing 50 mM Tris–HCl (pH 7.4 at 25°C), 140 mM KCl, 0.5 mM EDTA, MnCl_2_, CoCl_2_, NiCl_2_, ZnCl_2_, or CuCl_2_, 5 × SYPRO Orange dye (Sigma-Aldrich, USA), and 2 μM nsp15 was loaded into a 96-well PCR plate. The reaction was performed using a CFX Connect qPCR system. After an initial equilibration step at 25°C for 10 min, the temperature was increased by 0.5°C every 30 s until it reached 95 °C. During protein unfolding, SYPRO Orange dye binds to exposed hydrophobic regions nonspecifically, resulting in increased fluorescence intensity. Fluorescence intensity was measured after each cycle using the FRET channel. The final data were analyzed using Bio-Rad CFX Maestro software.

### Centrifugal analysis

20 μL of sample containing 50 mM Tris–HCl (pH 8.0 at 25°C), 100 mM NaCl, zero or 0.5 mM MnCl_2_, CoCl_2_, NiCl_2_, or ZnCl_2_, and 12 μM nsp15 was incubated at 37°C for 10 min. Then, 1 μL of H_2_O or 100 mM EDTA was added to the sample, followed by mixing. 5 μL of the sample was extracted for 12.5% SDS-PAGE. The remaining 16 μL of the sample was subjected to centrifugation at 11  300 × *g* and room temperature for 10 min. Subsequently, 5 μL of the supernatant was extracted for 12.5% SDS-PAGE.

### Negative-staining electron microscopy

A 3 µL sample of 0.03 mg/mL Strep-H234A, supplemented with 7.5 µM dsRNA44 and 5 mM CoCl_2_, NiCl_2_, or ZnCl_2_, was applied to a copper grid (Beijing XXBR Technology, no. T10023), incubated for 1 min, and absorbed with filter paper. The grid was then stained with 3 µL of 2% Uranium acetate for 1 min and air-dried. Images were acquired using a Talos L120C G2 120 kV transmission electron microscope.

### Cryo-EM imaging and data processing

In our structural studies, we utilized the recombinant protein with a Strep-Tag II of the catalytically impaired H234A mutant of SARS-CoV-2 nsp15, Strep-H234A, which was diluted to a final concentration of 7.5 µM. The nsp15/dsRNA complex sample was prepared by mixing Strep-H234A with dsRNA44 at a molar ratio of 1:10, followed by incubation on ice for 30 min in the presence of 5 mM Co^2+^. A 3 μL sample was applied to glow-discharged Quantifoil R1.2/1.3 300-mesh copper grid using a Vitrobot Mark IV (FEI) under the following conditions: blotting force of 3, blotting time of 3 s, 100% humidity, and a temperature of 8°C. The grid was then plunge-frozen in liquid ethane and transferred to liquid nitrogen for storage. Initial screening was conducted on an FEI Glacios microscope (Core Facility of Wuhan University) operating at 200 kV and equipped with a Ceta D CMOS camera. A grid exhibiting optimal ice thickness and particle distribution was selected for high-resolution cryo-EM data collection. Data acquisition was performed using an FEI Titan Krios G4 microscope (Core Facility of Wuhan University) operating at 300 kV and equipped with a Gatan K3 direct electron detector. For the nsp15/dsRNA complex, a total of 4 868 movies were collected using Thermo Scientific EPU software in counting mode. Each movie consisted of 40 frames, with a cumulative electron dose of 50 electrons per Å2 and a pixel size of 0.84 Å. All datasets were processed using CryoSPARC [35].

For the nsp15/dsRNA complex, the initial dataset underwent blob-picking followed by two rounds of 2D classification, yielding 319  161 particles for further processing. These particles were subjected to ab-initio reconstruction, followed by iterative rounds of homogeneous and heterogeneous refinement under C1 symmetry. The optimal class from this process was selected for additional homogeneous refinement with C1 symmetry, resulting in 244  604 particles. Subsequent global CTF refinement and additional homogeneous refinement were performed with C1 symmetry, followed by local CTF refinement and volume flipping. The flipped volume was then used for both homogeneous and non-uniform refinement under C1 symmetry, yielding a 6:1 nsp15/dsRNA map at 2.6 Å resolution. This 6:1 nsp15/dsRNA map was subsequently used for heterogeneous refinement under C1 symmetry. The best class obtained from this step underwent further homogeneous refinement under C1 symmetry. The refined volume was then combined with the initial 319  161 particles for another round of heterogeneous refinement. The optimal class from this final refinement step was subjected to homogeneous and non-uniform refinement under C1 symmetry, ultimately producing a 6:2 nsp15/dsRNA map at 2.78 Å resolution.

### Model building and structure refinement

The 7TJ2 structure served as the initial model for constructing the 6:1 nsp15/dsRNA complex, which was subsequently used to build the 6:2 nsp15/dsRNA complex. All models were generated using Coot [36]. Model refinement was performed through real-space refinement in PHENIX [37]. The overall model quality was evaluated using MolProbity [38]. Detailed statistical analyses are provided in Supplementary Table S3. Structural figures were prepared using PyMOL and UCSF ChimeraX [39].

## Results

### Co^2+^ and Ni^2+^ strongly activate SARS-CoV-2 nsp15

**Figure 1.**
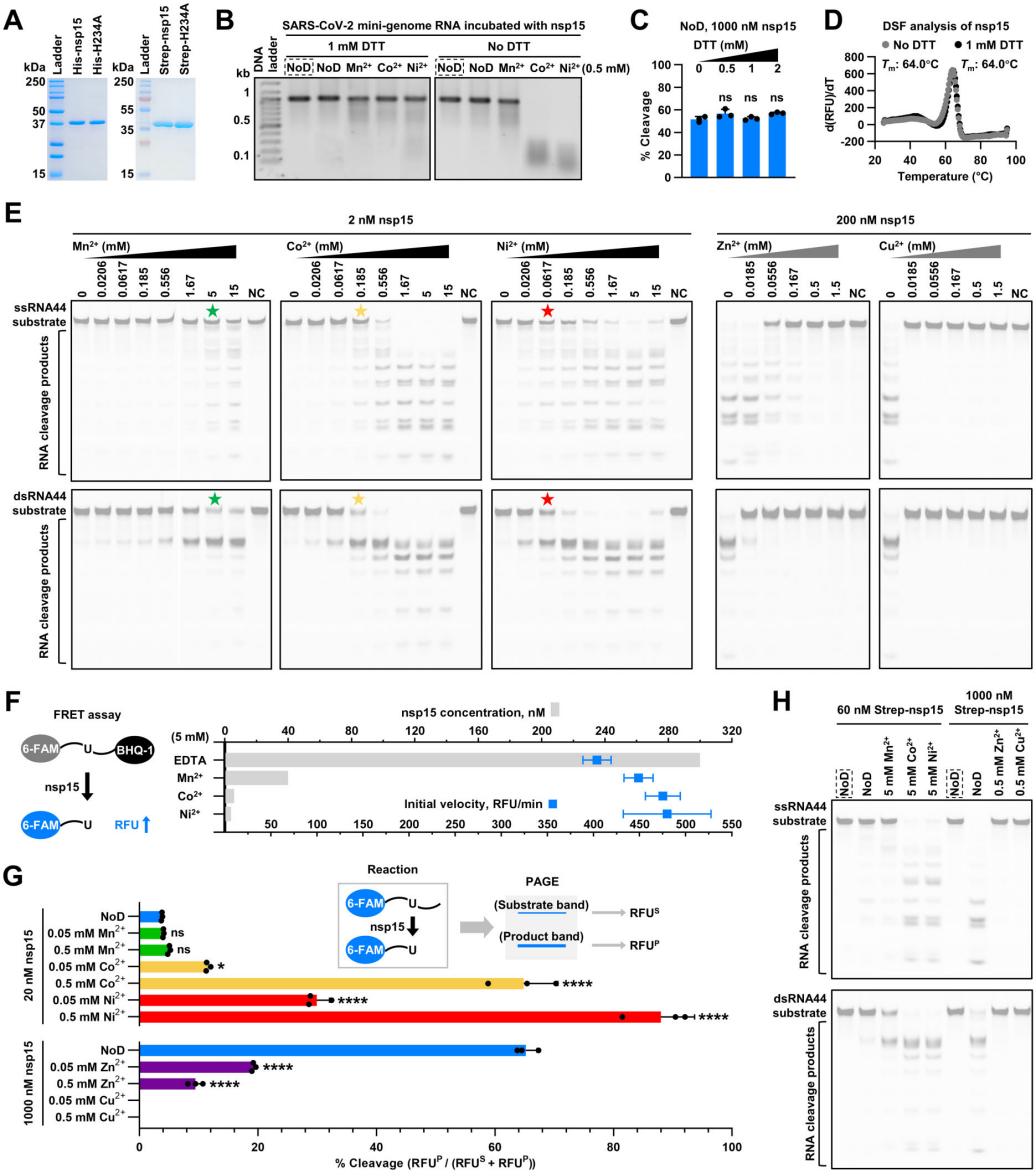
Co^2+^ and Ni^2+^ strongly facilitate, whereas Zn^2+^ and Cu^2+^ markedly impede RNA cleavage by SARS-CoV-2 nsp15. (**A**) SDS-PAGE analysis of purified ∼40 kDa recombinant SARS-CoV-2 nsp15 and its active-site mutant, H234A, both with a 6 × His tag (His-nsp15 and His-H234A) or a Strep-Tag II (Strep-nsp15 and Strep-H234A) at the N-terminus. Unless specifically indicated as Strep-nsp15, His-nsp15 was used in subsequent biochemical experiments. (**B**) Cleavage of the SARS-CoV-2 mini-genome RNA [16] by SARS-CoV-2 nsp15 under different conditions. NoD refers to no divalent cations. The reactions with the H234A mutant are marked with dashed boxes. (**C**) Quantification of the RNA cleavage efficiencies of SARS-CoV-2 nsp15 under various concentrations of DTT in the absence of divalent cations using the method described in (**G**). (**D**) DSF analysis of SARS-CoV-2 nsp15 with or without DTT in the absence of divalent cations. The peak in the d(RFU)/dT (first derivative of fluorescence intensity) versus temperature graph corresponds to the melting temperature (*T*_m_). (**E**) Cleavage of the ssRNA44 and dsRNA44 substrates by SARS-CoV-2 nsp15 under various concentrations of Mn^2+^, Co^2+^, Ni^2+^, Zn^2+^, or Cu^2+^. The reactions containing the H234A mutant and the highest concentration of various divalent cations were used as negative controls (NC). The RNA sequences are displayed in Supplementary Fig. S1. The RNA cleavage extents of the samples marked with pentagrams are similar, and these samples were used to identify the preferred cleavage sites of SARS-CoV-2 nsp15 in ssRNA44 and dsRNA44 (see Supplementary Fig. S1). (**F**) Quantification of the enhancing effects of 5 mM Mn^2+^, Co^2+^, and Ni^2+^ on SARS-CoV-2 nsp15 activity using a FRET assay (see also Supplementary Fig. S2). The average and standard deviation of three independent reactions are graphed. (**G**) Quantification of the effects of 0.05 and 0.5 mM Mn^2+^, Co^2+^, Ni^2+^, Zn^2+^, and Cu^2+^ on SARS-CoV-2 nsp15 activity using a single-U-containing substrate (see also Supplementary Fig. S3). In (C and G), the average and standard deviation of three independent reactions are graphed. Student’s *t*-test was performed with the RNA cleavage efficiency measured in the reaction with no DTT and no divalent cations as a control. ns, not significant, *P* > 0.05; *P < 0.05; ****P < 0.0001. (**H**) Cleavage of the ssRNA44 and dsRNA44 substrates by Strep-nsp15 under different conditions. The reactions with Strep-H234A are marked with dashed boxes.

To elucidate the role of metal ions in nsp15, we purified the recombinant proteins of SARS-CoV-2 nsp15 and its active-site mutant, H234A, both with an N-terminal 6 × His tag (Fig. 1A, left panel), and then systematically examined the effects of multiple divalent cations on them *in vitro*. Our previous study demonstrated that SARS-CoV-2 nsp15 prefers Mn^2+^ over Mg^2+^ and Ca^2+^ for promoting RNA cleavage [16], consistent with the studies on SARS-CoV nsp15 and MERS-CoV nsp15 [21–23]. In the present study, two additional ions, Co^2+^ and Ni^2+^, were tested. However, the dithiothreitol (DTT)-containing buffer, which was commonly employed in previous studies, could mask the effects of Co^2+^ and Ni^2+^ on nsp15, as these two ions appear to undergo reduction with DTT (Fig. 1B). We demonstrated that DTT has no significant effect on the activity and stability of SARS-CoV-2 nsp15 (Fig. 1C and D), and then investigated the effects of Co^2+^ and Ni^2+^ on nsp15 activity under DTT-free conditions. Surprisingly, it was observed that Co^2+^ and Ni^2+^ facilitated RNA cleavage by SARS-CoV-2 nsp15 much more strongly than Mn^2+^ (Fig. 1B and E), without a notable alteration in the cleavage specificity (Fig. 1E and Supplementary Fig. S1). A fluorescence resonance energy transfer (FRET) assay was utilized to quantify the enhancement at the optimal ion concentration. Different concentrations of nsp15 were employed under the conditions of 5 mM EDTA, Mn^2+^, Co^2+^, and Ni^2+^ to achieve a similar initial velocity of RNA cleavage, and the use of nsp15 under 5 mM EDTA was at least 75 times higher than under 5 mM Ni^2+^, 50 times higher than under 5 mM Co^2+^, and 7.5 times higher than under 5 mM Mn^2+^ (Fig. 1F and Supplementary Fig. S2). The quantification experiments using a single-U-containing substrate at low ion concentrations showed that 0.05 and 0.5 mM Mn^2+^ did not significantly increase nsp15 activity, whereas 0.05 and 0.5 mM Co^2+^ enhanced nsp15 activity 3-fold and 17-fold, and 0.05 mM and 0.5 mM Ni^2+^ increased nsp15 activity 8-fold and 23-fold (Fig. 1G and Supplementary Fig. S3). Given that nsp15 can catalyze both the transphosphorylation of RNA to form a 2′,3′-cyclic phosphodiester and its subsequent hydrolysis to a 3′-phosphomonoester [25, 34], we further investigated the effects of Co^2+^ and Ni^2+^ on the second step. The results demonstrated the pronounced enhancement of Co^2+^ and Ni^2+^ on the hydrolysis of 2′,3′-cyclic phosphodiesters by SARS-CoV-2 nsp15, which was also significantly stronger than that of Mn^2+^ (Supplementary Fig. S4).

### Zn^2+^ and Cu^2+^ markedly suppress SARS-CoV-2 nsp15 activity

We made a preliminary investigation into the effects of Zn^2+^ and Cu^2+^ on SARS-CoV-2 nsp15 activity in our previous study [16] and further studied it in this research. It was observed that both ions markedly impeded RNA cleavage by SARS-CoV-2 nsp15 (Fig. 1E). The inhibitory effects of these two ions were also quantified using the single-U-containing substrate, which indicated that the presence of 0.05 and 0.5 mM Zn^2+^ resulted in a 3-fold and 7-fold reduction of nsp15 activity, while the introduction of 0.05 and 0.5 mM Cu^2+^ led to the complete elimination of nsp15 activity (Fig. 1G and Supplementary Fig. S3). Furthermore, we observed that Zn^2+^ and Cu^2+^ also exhibited pronounced inhibition on the hydrolysis of 2′,3′-cyclic phosphodiesters by SARS-CoV-2 nsp15 (Supplementary Fig. S4). In order to ascertain whether the inhibition exerted by Zn^2+^ and Cu^2+^ is reversible, we initially incubated nsp15 with Zn^2+^ and Cu^2+^, subsequently removing these ions by the addition of excess EDTA, and then proceeded to test the transphosphorylation and hydrolysis activities of nsp15. The results indicated that following the removal of Zn^2+^, both activities of SARS-CoV-2 nsp15 were recovered, whereas the protein treated with Cu^2+^ did not display any observable activity after Cu^2+^ removal (Supplementary Fig. S3 and S4).

The affinity of transition metal ions (Zn^2+^, Cu^2+^, Co^2+^, and Ni^2+^, among others) for histidine has been reported for a long time and has been widely applied in immobilized metal ion affinity chromatography [40]. To eliminate the possibility that the observed effects of Zn^2+^, Cu^2+^, Co^2+^, and Ni^2+^ on SARS-CoV-2 nsp15 were due to interactions with the 6 × His tag of the recombinant nsp15 protein, a Strep-Tag II (Trp-Ser-His-Pro-Gln-Phe-Glu-Lys) was introduced in place of the 6 × His tag to obtain the high-purity recombinant nsp15 protein (Strep-nsp15) and its active-site mutant (Strep-H234A) (Fig. 1A, right panel). The effects of Zn^2+^, Cu^2+^, Co^2+^, and Ni^2+^ on Strep-nsp15 were tested. It was observed that Zn^2+^ and Cu^2+^ suppressed the RNA cleavage activity of Strep-nsp15, while Co^2+^ and Ni^2+^ enhanced it (Fig. 1H). These results suggest that Zn^2+^, Cu^2+^, Co^2+^, and Ni^2+^ function by targeting SARS-CoV-2 nsp15 itself.

### Co^2+^, Ni^2+^, and Zn^2+^ reduce the stability of SARS-CoV-2 nsp15, while Cu^2+^ denatures it

**Figure 2.**
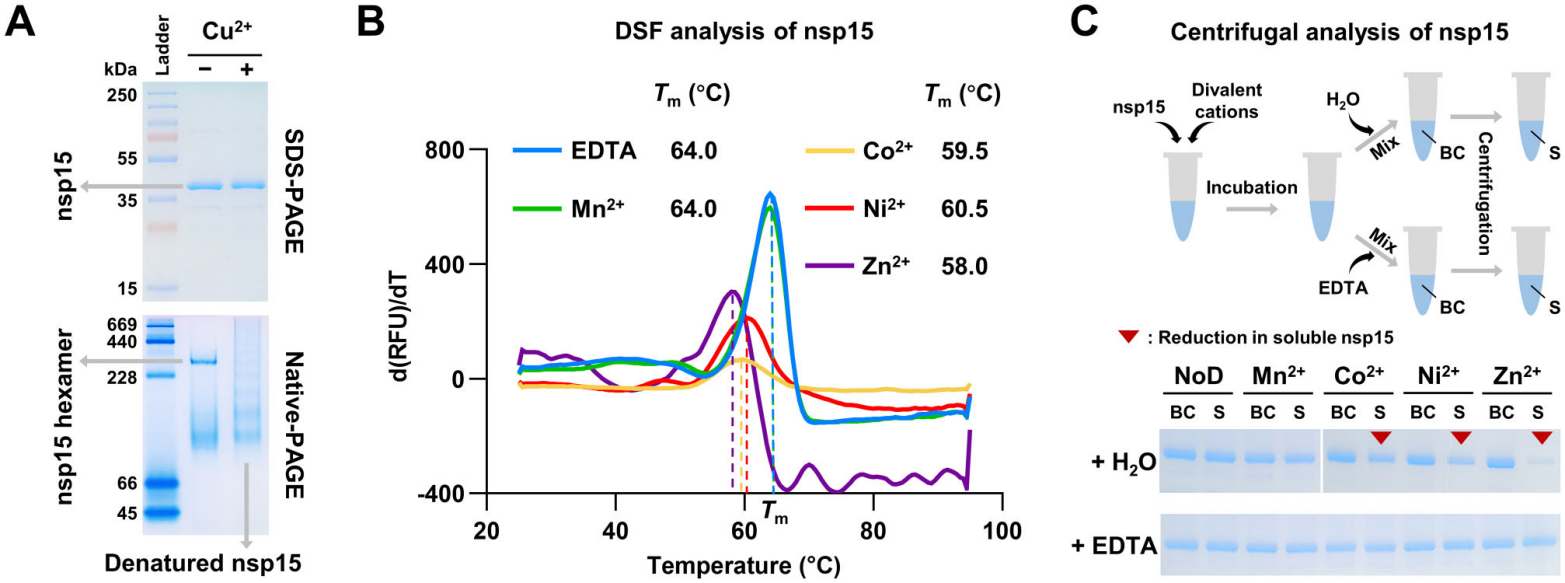
Co^2+^, Ni^2+^, and Zn^2+^ reduce the stability of SARS-CoV-2 nsp15, while Cu^2+^ denatures it. (**A**) SDS-PAGE and native-PAGE analysis of SARS-CoV-2 nsp15 protein with (+) or without (−) 0.5 mM Cu^2+^ treatment. (**B**) DSF analysis of SARS-CoV-2 nsp15 in the presence of 0.5 mM EDTA, Mn^2+^, Co^2+^, Ni^2+^, or Zn^2+^. (**C**) Centrifugal analysis of SARS-CoV-2 nsp15 under conditions of no divalent cations or with 0.5 mM Mn^2+^, Co^2+^, Ni^2+^, or Zn^2+^, or after removal of these ions by the addition of 5 mM EDTA. The samples before centrifugation (BC) and the subsequent supernatant (**S**) were analyzed by SDS-PAGE.

Considering the irreversible inhibition of SARS-CoV-2 nsp15 activity by Cu^2+^, we hypothesized that Cu^2+^ may denature it. We analyzed the nsp15 protein with or without Cu^2+^ treatment by Native-PAGE. The gel bands of the nsp15 protein treated with Cu^2+^ displayed an unusual ladder-like pattern, while the prominent band of the nsp15 protein without Cu^2+^ treatment was slightly higher than the 228 kDa marker, consistent with the size of the native hexameric form of SARS-CoV-2 nsp15 (approximately 240 kDa) (Fig. 2A). The SDS-PAGE analysis showed that the gel bands of the nsp15 protein with or without Cu^2+^ treatment exhibited no discernible difference (Fig. 2A). These results suggest that Cu^2+^ irreversibly alters the configuration of SARS-CoV-2 nsp15, with little effect on its primary structure.

Although Mn^2+^ is able to promote RNA cleavage by nsp15, the effect of Mn^2+^ on nsp15 stability appears to be subtle [16, 26, 32], suggesting that the interaction between Mn^2+^ and nsp15 may be non-structural. Given the pronounced effects of Co^2+^, Ni^2+^, and Zn^2+^ on SARS-CoV-2 nsp15 activity, we therefore explored their effects on SARS-CoV-2 nsp15 stability. Differential scanning fluorimetry (DSF) is routinely used to determine the melting temperature (*T*_m_) of proteins. We performed DSF analysis of SARS-CoV-2 nsp15 under conditions containing EDTA or selected divalent cations. The results indicated that the *T*_m_ of SARS-CoV-2 nsp15 with 0.5 mM Co^2+^, Ni^2+^, or Zn^2+^ was lower than that with 0.5 mM EDTA or Mn^2+^ (Fig. 2B), suggesting that Co^2+^, Ni^2+^, and Zn^2+^ destabilize SARS-CoV-2 nsp15. With the reduced nsp15 stability by Co^2+^, Ni^2+^, or Zn^2+^, we observed protein aggregation at a high nsp15 concentration, whereas this phenomenon was not observed either in the presence of Mn^2+^ or in the absence of divalent cations (Fig. 2C). Nevertheless, the protein aggregation was eliminated when Co^2+^, Ni^2+^, or Zn^2+^ was removed by further addition of EDTA (Fig. 2C), suggesting that the effects caused by these ions are reversible. Notably, Zn^2+^ exerted the most significant effect on SARS-CoV-2 nsp15 stability in comparison to Co^2+^ and Ni^2+^ (Fig. 2B and C), suggesting that the specific interaction of Zn^2+^ with SARS-CoV-2 nsp15 might be the most potent.

### Co^2+^ enhances binding between SARS-CoV-2 nsp15 and dsRNA

**Figure 3.**
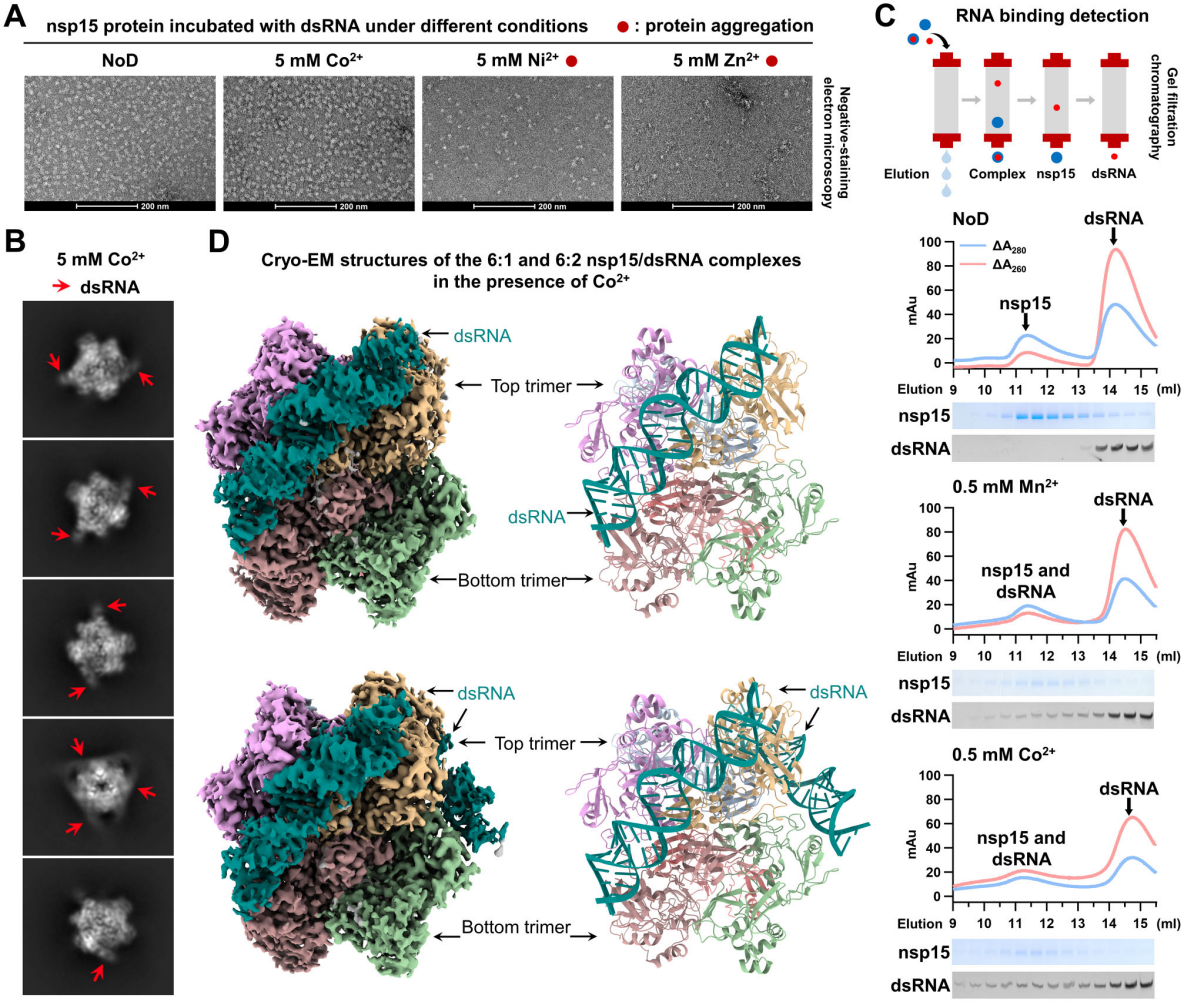
Co^2+^ enhances binding between SARS-CoV-2 nsp15 and dsRNA. (**A**) Representative negative-staining images of the H234A mutant in complex with dsRNA under conditions of no divalent cations or with 5 mM Co^2+^, Ni^2+^, or Zn^2+^. (**B**) 2D class averages of different SARS-CoV-2 nsp15/dsRNA complexes under 5 mM Co^2+^ condition. (**C**) Detecting RNA binding to the H234A mutant under conditions of no divalent cations or with 0.5 mM Mn^2+^ or Co^2+^ by gel filtration chromatography. (**D**) Cryo-EM density maps (left) and ribbon diagrams (right) of the 6:1 and 6:2 SARS-CoV-2 nsp15/dsRNA complexes in the presence of Co^2+^.

Despite the efforts in structural studies, the metal-binding sites had not been identified for nsp15, with the use of Mn^2+^ in the majority of them [24, 33, 34]. Given the significant effects of Co^2+^, Ni^2+^, and Zn^2+^ on SARS-CoV-2 nsp15 activity and stability, we attempted to capture the SARS-CoV-2 nsp15 structures in complex with dsRNA in the presence of Co^2+^, Ni^2+^, or Zn^2+^ using cryo-EM, aiming to determine the metal-binding sites of nsp15. In order to prevent dsRNA cleavage and potential interactions between these three ions and the 6 × His tag, we used the nsp15 active-site mutant with a Strep-Tag II, Strep-H234A. We incubated the nsp15 protein with dsRNA in the presence of 5 mM Co^2+^, Ni^2+^, or Zn^2+^. Both Zn^2+^ and Ni^2+^ induced severe protein aggregation (Fig. 3A), consistent with the observation in the centrifugal analysis (Fig. 2C), thus preventing the continuation of the structural studies with Zn^2+^ and Ni^2+^. We carried out cryo-EM imaging on the sample with Co^2+^. Notably, unlike previously reported cryo-EM 2D class averages of SARS-CoV-2 nsp15 in complex of dsRNA that predominantly showed only one dsRNA bound per nsp15 hexamer [41, 42], the 2D class averages of the sample with Co^2+^ in our study revealed different nsp15/dsRNA complexes with up to three dsRNAs bound to one nsp15 hexamer (Fig. 3B). Considering that Co^2+^ was not used in previous structural studies, with no divalent cations or 5 mM Mn^2+^ present in the cryo-EM sample, we hypothesized that Co^2+^ is capable of enhancing binding between SARS-CoV-2 nsp15 and dsRNA. To confirm the enhancement, we initially adopted electrophoretic mobility shift assay (EMSA). However, no RNA binding to nsp15 was detected when analyzing the samples either with no divalent cations or with 5 mM Mn^2+^, Co^2+^, Ni^2+,^ or Zn^2+^ (Supplementary Fig. S5). We speculated that during electrophoresis, the samples are unable to consistently maintain specific ion conditions, which may affect the interaction between divalent cations and nsp15. Therefore, we employed gel filtration chromatography to perform RNA binding detection, which can keep nsp15 and divalent cations together throughout (Fig. 3C). In the absence of divalent cations, no discernible interaction was observed between nsp15 and dsRNA, and nsp15 and dsRNA were eluted separately, with nsp15 eluting early and dsRNA eluting late (Fig. 3C). The presence of 0.5 mM Mn^2+^ resulted in the elution of some dsRNA in conjunction with nsp15, and this phenomenon was more pronounced in the presence of 0.5 mM Co^2+^ (Fig. 3C). These results suggest that both Mn^2+^ and Co^2+^ are capable of enhancing the interaction between SARS-CoV-2 nsp15 and dsRNA, with Co^2+^ exhibiting superior efficacy.

### Cryo-EM structures of SARS-CoV-2 nsp15/dsRNA complexes with Co^2+^ reveal extra density at the active site

**Figure 4.**
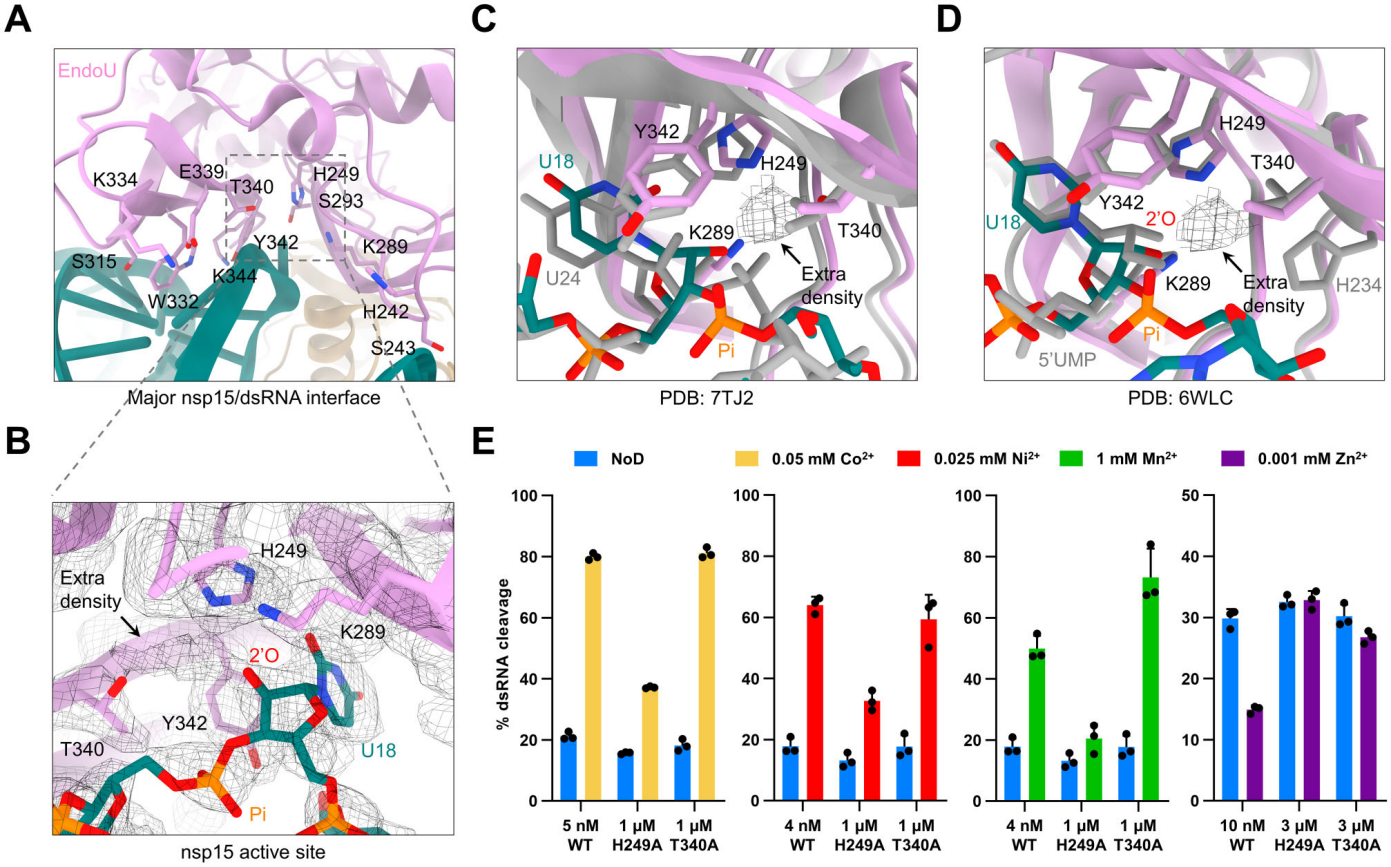
Active-site mutation H249A weakens the effects of Co^2+^, Ni^2+^, Mn^2+^, and Zn^2+^ on SARS-CoV-2 nsp15. (**A**) Enlarged view of the major nsp15/dsRNA interface formed by the EndoU domain (plum). (**B**) Cryo-EM density validation of the nsp15 active site. The residues (plum) around the extra density (marked by a black arrow) and the flipped-out U18 from the bound dsRNA (teal) are shown. The cryo-EM density is shown at a contour level of 0.36. (**C**) Enlarged view of the overlaid ribbon diagram, showing the conformational changes of the previously reported nsp15/dsRNA complex (PDB 7TJ2; gray) to our nsp15/dsRNA complex (colored). The extra density observed in our structure is shown and marked by a black arrow. (**D**) Enlarged overlaid ribbon diagram of the nsp15 active site between the UMP-bound nsp15 complex (PDB 6WLC; gray) and our nsp15/dsRNA complex (colored). The extra density observed in our structure is shown and marked by a black arrow. (**E**) Quantification of the effects of mutations at H249 and T340, residues near the extra density, on the regulation of SARS-CoV-2 nsp15 activity by Co^2+^, Ni^2+^, Mn^2+^, and Zn^2+^ (see also Supplementary Fig. S11). The average and standard deviation of three independent reactions are graphed.

Among all SARS-CoV-2 nsp15/dsRNA complexes, we successfully obtained high-resolution structures for those containing one (6:1 nsp15/dsRNA complex with an overall resolution of 2.6 Å) and two (6:2 nsp15/dsRNA complex with a resolution of 2.78 Å) dsRNA molecules (Fig. 3D and Supplementary Fig. S6). In both complexes, nsp15 forms a hexamer composed of two back-to-back trimers with clear densities of bound dsRNA. Each dsRNA engages three of the six nsp15 protomers, forming two distal interfaces and the major interface at the cleavage site of the dsRNA (Supplementary Fig. S7A and S8). The first distal interface (Interface I) is mediated by one of the top nsp15 protomers and involves K12 and Q18 in the middle domain (MD) as well as S147 in the N-terminal domain (NTD) (Supplementary Fig. S7B and S8B). The second distal interface (Interface II) is mediated by one of the bottom nsp15 protomers and involves K110, T112, R135, and N136 in the MD (Supplementary Fig. S7C and S8C). The interactions at the major interface (Interface III) are mediated by the catalytic EndoU domain of another top nsp15 protomer, and the residues that interact with dsRNA are analogous to those previously reported [41, 42], including H242, S243, H249, K289, S293, S315, W332, K334, E339, T340, Y342, and K344 (Fig. 4A and Supplementary Fig. S8D). Compared with the apo nsp15 structures obtained by X-ray crystallography [32], the bottom trimer of nsp15 complex rotates toward the bound dsRNA (Supplementary Fig. S9).

Intriguingly, we observed extra density at the active site of each nsp15 protomer in the nsp15/dsRNA complexes (Fig. 4B). Notably, in those protomers where the EndoU domain interacts with dsRNA, the extra density is significantly higher compared to the other protomers. Structural alignment of the previously reported nsp15/dsRNA complex [41] and our nsp15/dsRNA complex revealed an inward shift of the phosphate between the flipped-out U18 and A19 (Fig. 4C). This conformational change supports the extra density at the active site or otherwise the 3′ phosphate of the U18 would clash with the extra density. H249, K289, T340, and Y342, as well as the 2′ hydroxyl of the flipped-out uridine from dsRNA are in proximity to the extra density in our structure (Fig. 4B). By overlaying the UMP-bound nsp15 complex [24] with our structure, we found that H234, which was mutated to alanine in our structure, is also close to the extra density (Fig. 4D). However, the resolution of our structure precludes unambiguous assignment of the extra density as Co^2+^ or accurate modeling of its coordinating residues.

### Active-site mutation H249A weakens the effects of Co^2+^, Ni^2+^, Mn^2+^, and Zn^2+^ on SARS-CoV-2 nsp15

To investigate whether the residues surrounding the extra density are associated with the activating effect of Co^2+^, we created the H249A, K289A, T340A, and Y342A mutants (Supplementary Fig. S10) and tested the dsRNA cleavage activity of these nsp15 mutants in the absence or presence of Co^2+^. The attempt to quantify the activation of the K289A and Y342A mutants induced by Co^2+^ was unsuccessful due to the RNA cleavage by them in the absence of Co^2+^ being too weak to detect, even in a high-protein concentration, similar to the H234A mutant (Supplementary Fig. S11). Although mutation of H249 also significantly diminished the ability of SARS-CoV-2 nsp15 to cleave dsRNA, which is reflected by a marked increase in the nsp15 concentration required to achieve a similar RNA cleavage extent, the activation of the H249A mutant by Co^2+^ was successfully quantified and was found considerably less pronounced in comparison to the wild-type (WT) nsp15 (Fig. 4E). In contrast, mutation of T340, which also led to a substantial decrease in SARS-CoV-2 nsp15 activity, does not change the effect of Co^2+^ (Fig. 4E). These results suggest that H249 plays a role in the activation of SARS-CoV-2 nsp15 by Co^2+^.

We also examined the effects of Ni^2+^, Mn^2+^, and Zn^2+^ on the RNA cleavage activity of the H249A and T340A mutants. Similar to Co^2+^, Ni^2+^ had a less pronounced enhancing effect on the activity of the H249A mutant than on wild-type SARS-CoV-2 nsp15, whereas its effect on the T340A mutant was comparable to that on the wild-type (Fig. 4E). The enhancing effect of Mn^2+^ on the activity of the H249A mutant was also attenuated compared to wild-type SARS-CoV-2 nsp15 (Fig. 4E). Surprisingly, mutation of T340 significantly strengthened the enhancing effect of Mn^2+^ on nsp15 activity (Fig. 4E). In addition, both mutations, H249A and T340A, led to a reduction in the inhibitory effect of Zn^2+^ on SARS-CoV-2 nsp15 activity (Fig. 4E). These results suggest that while H249 is important for the effects of Ni^2+^, Mn^2+^, and Zn^2+^ on SARS-CoV-2 nsp15, T340 also involves the interactions with Mn^2+^ and Zn^2+^.

Given that Zn^2+^ exerted the most significant effect on SARS-CoV-2 nsp15 stability in comparison to Mn^2+^, Co^2+,^ and Ni^2+^ (Fig. 2B and C and Fig. 3A), it was hypothesized that the binding affinity of Zn^2+^ to nsp15 might be the highest. We examined the activation of SARS-CoV-2 nsp15 by Mn^2+^, Co^2,+^ or Ni^2+^ in the presence of Zn^2+^. It was observed that the addition of 0.05 mM Ni^2+^, 0.5 mM Co^2+^, or 5 mM Mn^2+^ under 0.05 mM Zn^2+^ did not result in an increase in the RNA cleavage activity of SARS-CoV-2 nsp15, whereas SARS-CoV-2 nsp15 could be strongly activated under these ion conditions without Zn^2+^ (Supplementary Fig. S12). These results indicated that the activating effects of Mn^2+^, Co^2+^, and Ni^2+^ on SARS-CoV-2 nsp15 could be completely counteracted by the inhibitory effect of Zn^2+^, suggesting competitive regulation.

### Specific effects of Co^2+^, Ni^2+^, and Zn^2+^ on nsp15 are widespread in coronaviruses

**Figure 5.**
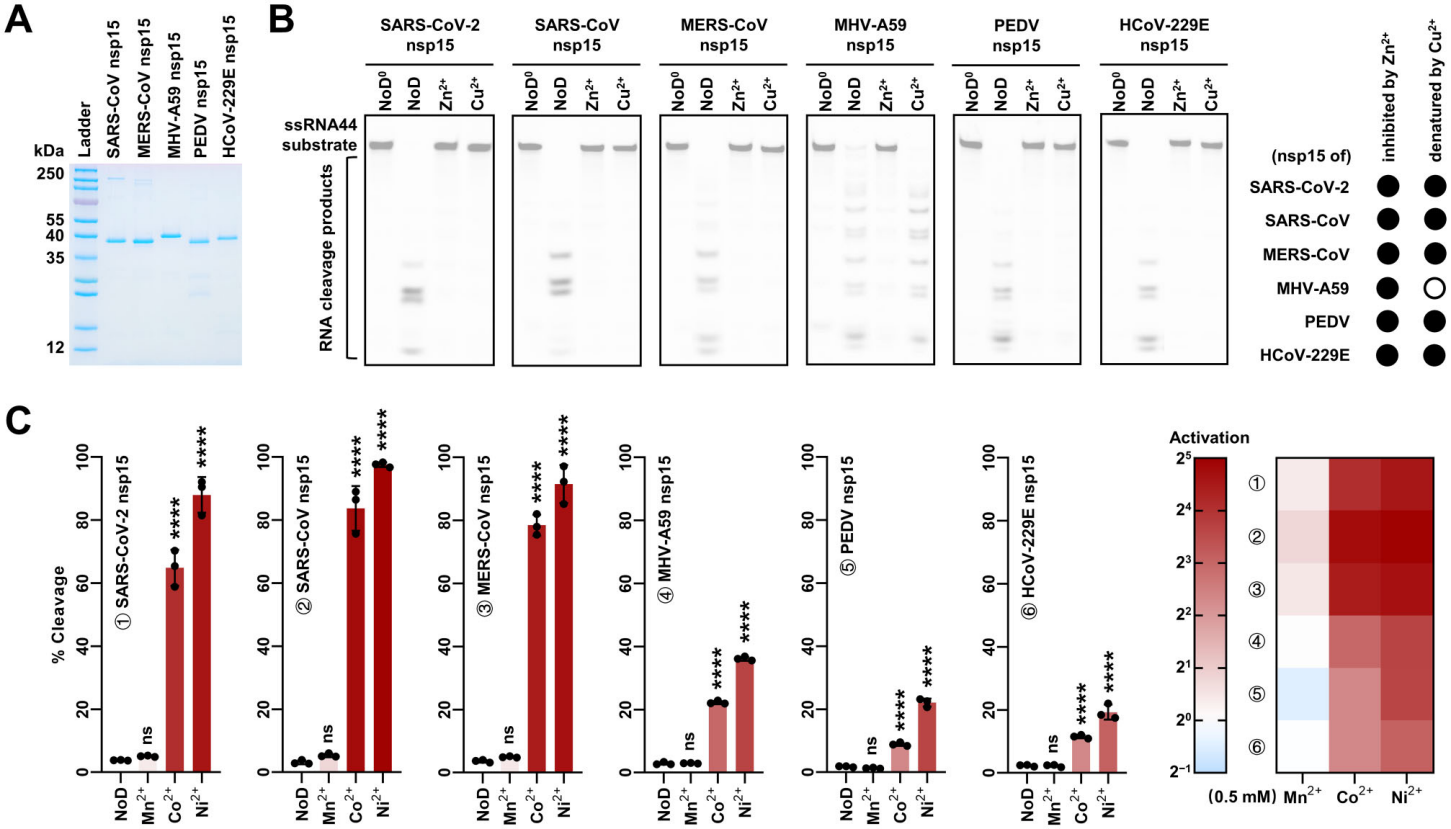
Specific effects of Co^2+^, Ni^2+^, and Zn^2+^ on nsp15 are widespread in coronaviruses. (**A**) SDS-PAGE analysis of purified recombinant nsp15 of various coronaviruses. (**B**) Cleavage of the ssRNA44 substrate by 300 nM nsp15 of various coronaviruses under conditions of no divalent cations or with 0.5 mM Zn²⁺ or Cu^2+^. The reactions lacking nsp15 are marked with ^0^. The effects of Zn^2+^ and Cu^2+^ on nsp15 of various coronaviruses are summarized in the right panel. (**C**) Quantification of the effects of 0.5 mM Mn^2+^, Co^2+^, and Ni^2+^ on the EndoU activity of nsp15 of various coronaviruses using the method described in Fig. 1G. The concentrations of SARS-CoV-2 nsp15, SARS-CoV nsp15, MERS-CoV nsp15, MHV-A59 nsp15, PEDV nsp15, and HCoV-229E nsp15 used in the experiment were 20, 30, 175, 225, 20, and 25 nM, respectively. The average and standard deviation of three independent reactions are graphed. Student’s *t-*test was performed with the RNA cleavage efficiency measured in the reaction with no divalent cations as a control. ns, not significant, *P* > 0.05; ****P < 0.0001. The fold increase or decrease in nsp15 activity is shown by a heatmap on the right panel.

To ascertain whether Mn^2+^, Co^2+^, Ni^2+^, Zn^2+^, and Cu^2+^ exert the same effects on the nsp15 of other coronaviruses as they do on SARS-CoV-2 nsp15, we purified the recombinant nsp15 of SARS-CoV, MERS-CoV, mouse hepatitis virus (MHV)-A59, porcine epidemic diarrhea virus (PEDV), and human coronavirus (HCoV)-229E (Fig. 5A and Supplementary Fig. S13), and investigated the effects of these ions on them. It was observed that in the absence of divalent cations, all of these nsp15 exhibited RNA cleavage activity (Fig. 5B), indicating that this activity is not dependent on divalent cations. In the presence of 0.5 mM Zn^2+^, the RNA cleavage activity of all nsp15 was markedly suppressed (Fig. 5B). Following the removal of Zn^2+^ by EDTA addition, the RNA cleavage activity of SARS-CoV nsp15, MHV-A59 nsp15, and HCoV-229E nsp15 was fully restored, whereas there was still a partial loss in the RNA cleavage activity of MERS-CoV nsp15 and PEDV nsp15, which may be due to the higher binding affinity of Zn^2+^ to these two proteins (Supplementary Fig. S14). These results suggest that the inhibitory effect of Zn^2+^ on nsp15 is a prevalent feature among coronaviruses. In addition, the RNA cleavage activity of the majority of these nsp15 was irreversibly eliminated by Cu^2+^, whereas Cu^2+^ demonstrated no significant effect on MHV-A59 nsp15 activity (Fig. 5B and Supplementary Fig. S14).

The effects of 0.5 mM Mn^2+^, Co^2+^, and Ni^2+^ on the EndoU activity of nsp15 of these coronaviruses were quantified using the single-U-containing substrate. As observed in SARS-CoV-2 nsp15, the activation of SARS-CoV nsp15 and MERS-CoV nsp15 by 0.5 mM Mn^2+^ was not significant, whereas 0.5 mM Co^2+^ and Ni^2+^ resulted in a 27-fold and 31-fold increase in SARS-CoV nsp15 activity, and a 22-fold and 26-fold increase in MERS-CoV nsp15 activity (Fig. 5C). Although the activation of MHV-A59 nsp15, PEDV nsp15, and HCoV-229E nsp15 by Co^2+^ and Ni^2+^ was less pronounced compared to the above three coronavirus nsp15, 0.5 mM Co^2+^ and Ni^2+^ resulted in an 8-fold and 13-fold increase in MHV-A59 nsp15 activity, a 5-fold and 12-fold increase in PEDV nsp15 activity, and a 5-fold and 8-fold increase in HCoV-229E nsp15 activity (Fig. 5C). In contrast, 0.5 mM Mn^2+^ had a negligible effect on the activity of MHV-A59 nsp15 and HCoV-229E nsp15, and even inhibited PEDV nsp15 activity, although this inhibition was not significant (Fig. 5C). These results suggest that the activation of Co^2+^ and Ni^2+^ on nsp15 is widespread in coronaviruses and predominantly potent, unlike the case of Mn^2+^.

Given the similarity between nsp15 and RNase A in terms of the catalytic mechanism as well as the active site [23, 31, 34, 43, 44], we investigated whether Co^2+^, Ni^2+^, and Zn^2+^ exert similar effects on RNase A as they do on nsp15. It was observed that Co^2+^ and Ni^2+^ did not enhance, but rather slightly inhibited RNase A activity (Supplementary Fig. S15). Zn^2+^ demonstrated a modest inhibitory effect on RNase A activity, which was significantly weaker than its effect on nsp15 (Supplementary Fig. S15). These results suggest that nsp15 has evolved specific interplay with Co^2+^, Ni^2+^, and Zn^2+^.

## Discussion

The regulatory mechanism of nsp15 activity is crucial in coronaviral replication. It is essential to guarantee that viral RNA cannot be degraded by nsp15 during genome replication and transcription, while residual viral dsRNA must be efficiently degraded by nsp15 to avoid being detected by host dsRNA sensors following the completion of genome replication and transcription. Based on our findings, we hypothesize that local concentrations of metal ions in different cellular contexts may govern nsp15 activity. Specifically, when nsp15 adopts a Zn^2+^-bound conformation, its endoribonuclease activity is suppressed to prevent degradation of viral RNA and allow viral genome replication and transcription, whereas when nsp15 is in Co^2+^/Ni^2+^-bound form, viral dsRNA is efficiently removed to prevent host immune activation. It is noteworthy that the structural studies of SARS-CoV-2 RNA-dependent RNA polymerase (nsp12), helicase (nsp13), and exoribonuclease (nsp14), as well as nsp10, the cofactor for nsp14 and nsp16, have revealed that Zn^2+^ plays a structural role in maintaining the correct conformation of these nsps [45–49]. These findings align with our hypothesis that nsp15 adopts a Zn^2+^-bound conformation during genome replication and transcription, in consideration of the co-localization of nsp15 and viral RTCs [9, 17–19].

However, the physiological relevance of Zn^2+^, Co^2+^, and Ni^2+^ in coronaviral replication remains to be proved. The intracellular concentrations of free Zn^2+^, Co^2+^, and Ni^2+^ are generally maintained at nanomolar or lower levels [50–52], which is significantly lower than what we used in the biochemical experiments. However, the local concentrations of these ions in specific cellular context during coronaviral replication are unclear and hard to determine due to complexity of the process. Interestingly, a recent study revealed extensive alterations in metal homeostasis, including zinc, cobalt, and nickel, in the body during the course of SARS-CoV-2 infection [53]. This suggests that zinc, cobalt, and nickel homeostasis might play a role in coronaviral replication. Moreover, zinc homeostasis has been demonstrated to be crucial for host innate immunity [54–59]. It is possible that coronaviruses employ zinc homeostasis to regulate their replication as a strategy to respond to host immunity. Therefore, the function of the transporters of zinc, cobalt, and nickel, such as zinc transporters and Zrt/Irt-related proteins [60, 61], in coronaviral replication is worthy of exploration.

In addition, in accordance with our hypothesis, interference with the regulation of nsp15 activity, whether its activation or inhibition, has the potential to affect coronaviral replication. Accordingly, Zn^2+^, Co^2+^, and Ni^2+^ or other regulators might be used to treat coronavirus disease. Actually, zinc has been proposed as a potential therapeutic agent for the treatment of COVID-19 due to its immunomodulatory and antiviral properties, and zinc therapy has been demonstrated to exhibit beneficial effects on patients infected with SARS-CoV-2 [62–65]. Our findings suggest that the effectiveness of zinc therapy is attributable not only to its role in mediating host antiviral immune response, but also potentially to its direct action on viral proteins. Notably, a recent study reported a guanidine-based coronavirus replication inhibitor which binds to the nsp15 active site, enhancing nsp15 activity and reducing nsp15 stability [66]. This work demonstrated the potential of the activator of nsp15 to suppress coronaviral replication.

## Supplementary Material

gkaf1508_Supplemental_File

## Data Availability

The cryo-EM maps for the 6:1 nsp15/dsRNA and 6:2 nsp15/dsRNA have been deposited in the Electron Microscopy Data Bank (EMDB) under accession codes EMDB-63618 and EMDB-63619, respectively. The cryo-EM coordinates for the 6:1 nsp15/dsRNA and 6:2 nsp15/dsRNA have been deposited in the Protein Data Bank (PDB) under accession codes 9M48 and 9M49, respectively. All other data are available in the manuscript and its supplementary data.
