# Supplementary material for: Metal ions govern coronavirus endoribonuclease activity
Source: Nucleic Acids Res. 2026 Jan 14;54(2):gkaf1508. doi: 10.1093/nar/gkaf1508 (PMC12802955; doi:10.1093/nar/gkaf1508)
Supplement: gkaf1508_Supplemental_File [file gkaf1508_supplemental_file.pdf]

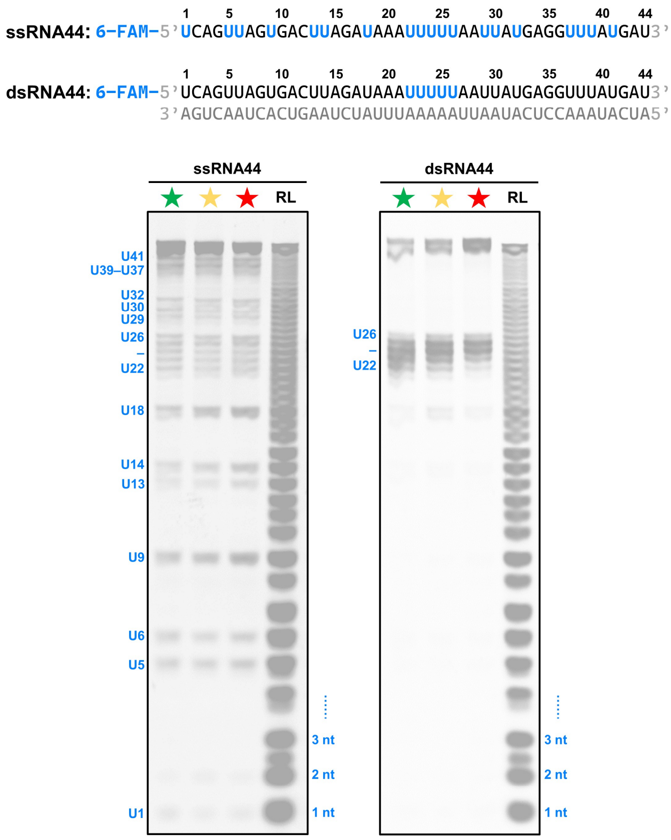

Supplementary Figure S1. Identification of the preferred cleavage sites of SARS-CoV-2 nsp15 in ssRNA44 and dsRNA44. The sequences of ssRNA44 and dsRNA44 are shown and the identified preferred cleavage sites of SARS-CoV-2 nsp15 in ssRNA44 and dsRNA44 are indicated in blue. The samples marked with pentagrams here correspond to the samples marked with the same pentagrams in Fig. 1E. RL refers to RNA ladder, which was generated by alkaline hydrolysis of ssRNA44. Regardless of whether it is under Mn^{2+} conditions or in the presence of Co^{2+} or Ni^{2+}, SARS-CoV-2 nsp15 preferentially cleaved the consecutive Us of an AU-rich area in dsRNA44 and cleaved almost all U sites in ssRNA44, although the cleavage efficiencies at these sites exhibited slight variations. This is consistent with our previous study on the cleavage preference of SARS-CoV-2 nsp15 (Ref.17).

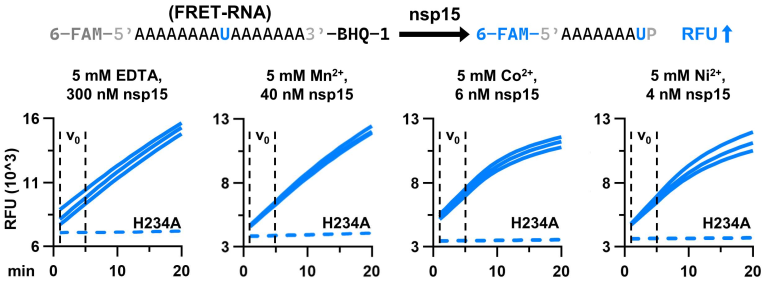

Supplementary Figure S2. Cleavage of the FRET-RNA substrate by SARS-CoV-2 nsp15 in the presence of 5 mM EDTA, Mn^{2+}, Co^{2+}, or Ni^{2+}, related to Fig. 1F. The cleavage of the FRET-RNA substrate would result in an increase in fluorescence. The initial velocity (v_0) was estimated using the slope from linear regressions of the reaction monitored during 1–5 min. The reactions with the active-site mutant of SARS-CoV-2 nsp15, H234A, were used as negative controls.

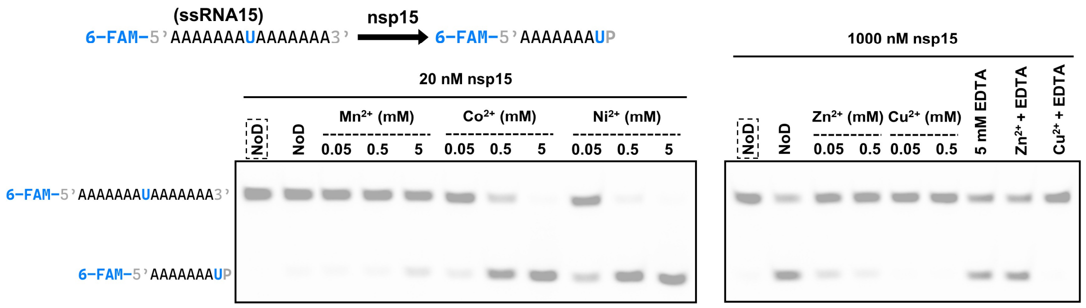

Supplementary Figure S3. Cleavage of the ssRNA15 substrate by SARS-CoV-2 nsp15 under different conditions, related to Fig. 1G. NoD refers to no divalent cations. Zn^{2+} + EDTA and Cu^{2+} + EDTA refer to that 0.5 mM Zn^{2+} or Cu^{2+} was removed by the addition of 5 mM EDTA after incubation with the nsp15 protein and before substrate addition. The reactions with the H234A mutant are marked with dashed boxes.

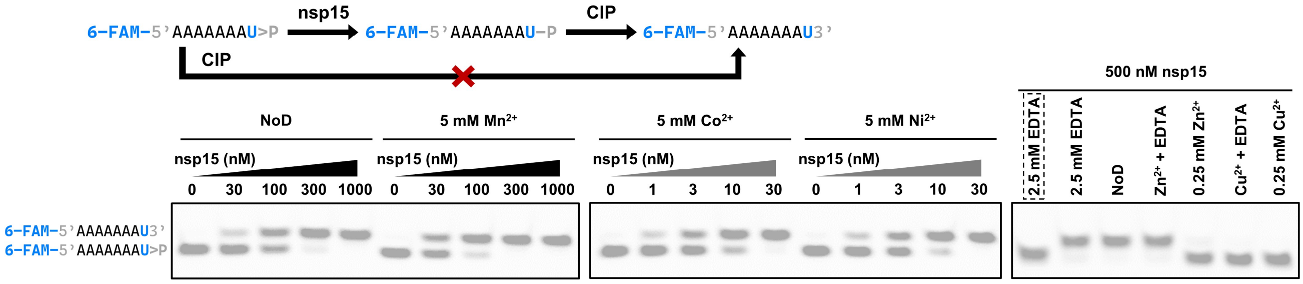

Supplementary Figure S4. Hydrolysis of 2',3'-cyclic phosphodiesters by SARS-CoV-2 nsp15 under different conditions. The RNA substrate containing a 2',3'-cyclic phosphate end was produced from ssRNA15 by RNase A. The RNA end can be identified as either a 2',3'-cyclic phosphate or a 3' phosphate by observing the change in electrophoretic mobility after the treatment of calf intestine alkaline phosphatase (CIP), which can only remove the 3' phosphate, leading to the loss of a negative charge. NoD refers to no divalent cations. Zn^{2+} + EDTA and Cu^{2+} + EDTA refer to that 0.25 mM Zn^{2+} or Cu^{2+} was removed by the addition of 2.5 mM EDTA after incubation with the nsp15 protein and before substrate addition. The reaction with the H234A mutant is marked with a dashed box.

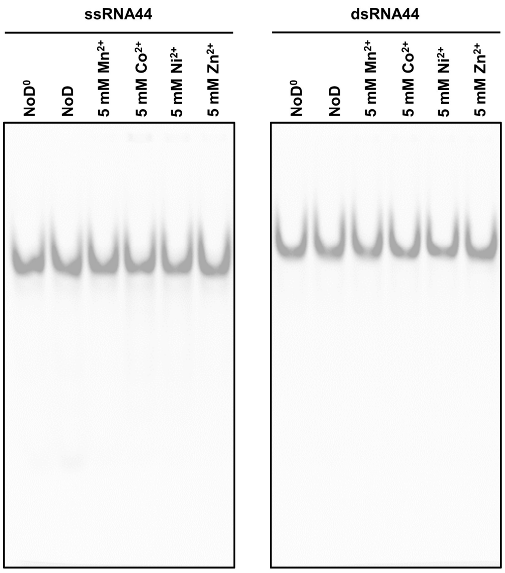

Supplementary Figure S5. EMSA analyzing the nsp15/RNA samples with no divalent cations (NoD) or 5 mM Mn^{2+}, Co^{2+}, Ni^{2+}, or Zn^{2+}. To prevent RNA cleavage, the H234A mutant were used in the experiment. The samples lacking the protein are marked with ^{0}.

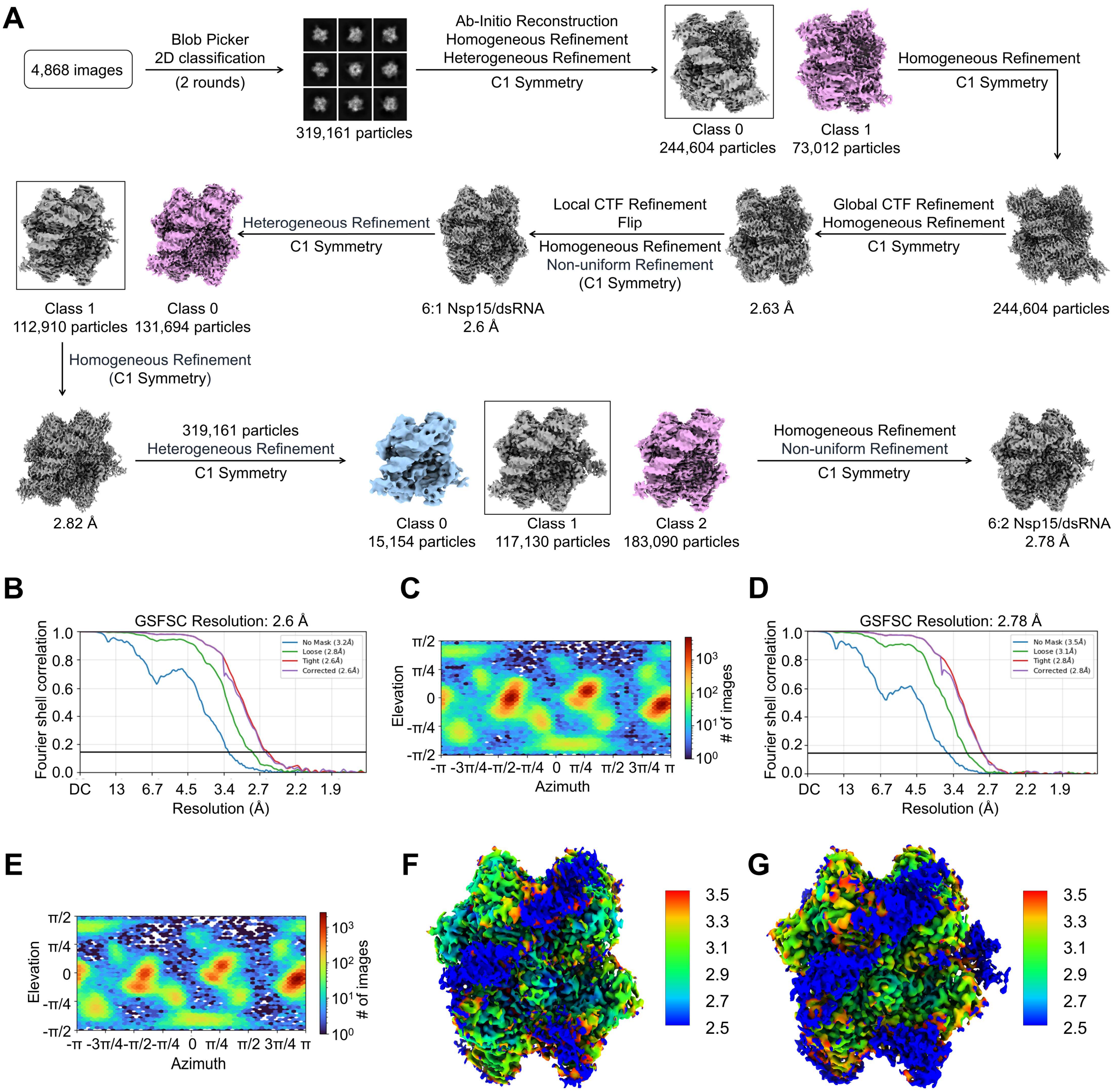

Supplementary Figure S6. Reconstruction of the 6:1 and 6:2 nsp15/dsRNA complexes. (**A**) Data processing workflow for the reconstruction of the 6:1 and 6:2 nsp15/dsRNA complexes. (**B**) Gold-standard FSC curves of the 6:1 nsp15/dsRNA complex indicate an overall resolution of 2.6 Å at FSC=0.143. (**C**) The orientation distribution plot of the 3D reconstruction of the 6:1 nsp15/dsRNA complex. (**D**) Gold-standard FSC curves of the 6:2 nsp15/dsRNA complex indicate an overall resolution of 2.78 Å at FSC=0.143. (**E**) The orientation distribution plot of the 3D reconstruction of the 6:2 nsp15/dsRNA complex. (**F**) Local resolution map of the 6:1 nsp15/dsRNA complex. (**G**) Local resolution map of the 6:2 nsp15/dsRNA complex.

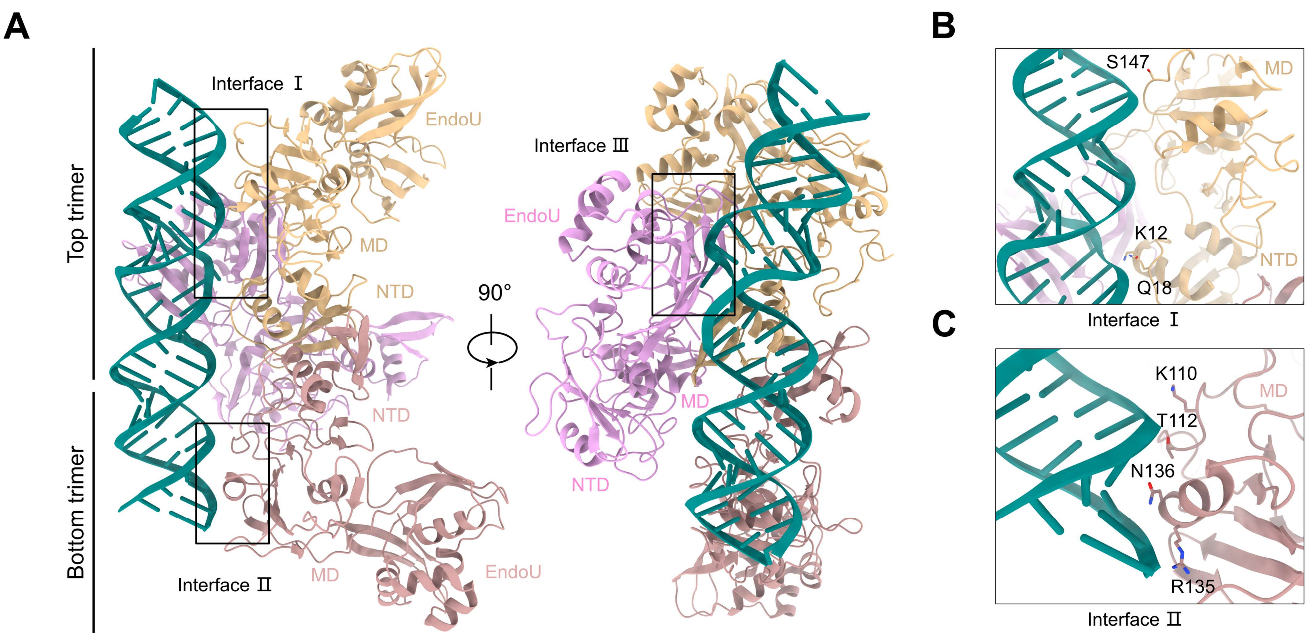

Supplementary Figure S7. Ribbon diagrams of the dsRNA binding interfaces in the nsp15/dsRNA complex. (**A**) Ribbon diagram of the dsRNA binding interfaces in two orthogonal views. The dsRNA engages three of the six nsp15 protomers. (**B**) Enlarged view of the first distal interface formed by the NTD and MD domains of nsp15 (burly wood). (**C**) Enlarged view of the second distal interface formed by the MD domain of nsp15 (rosy brown).

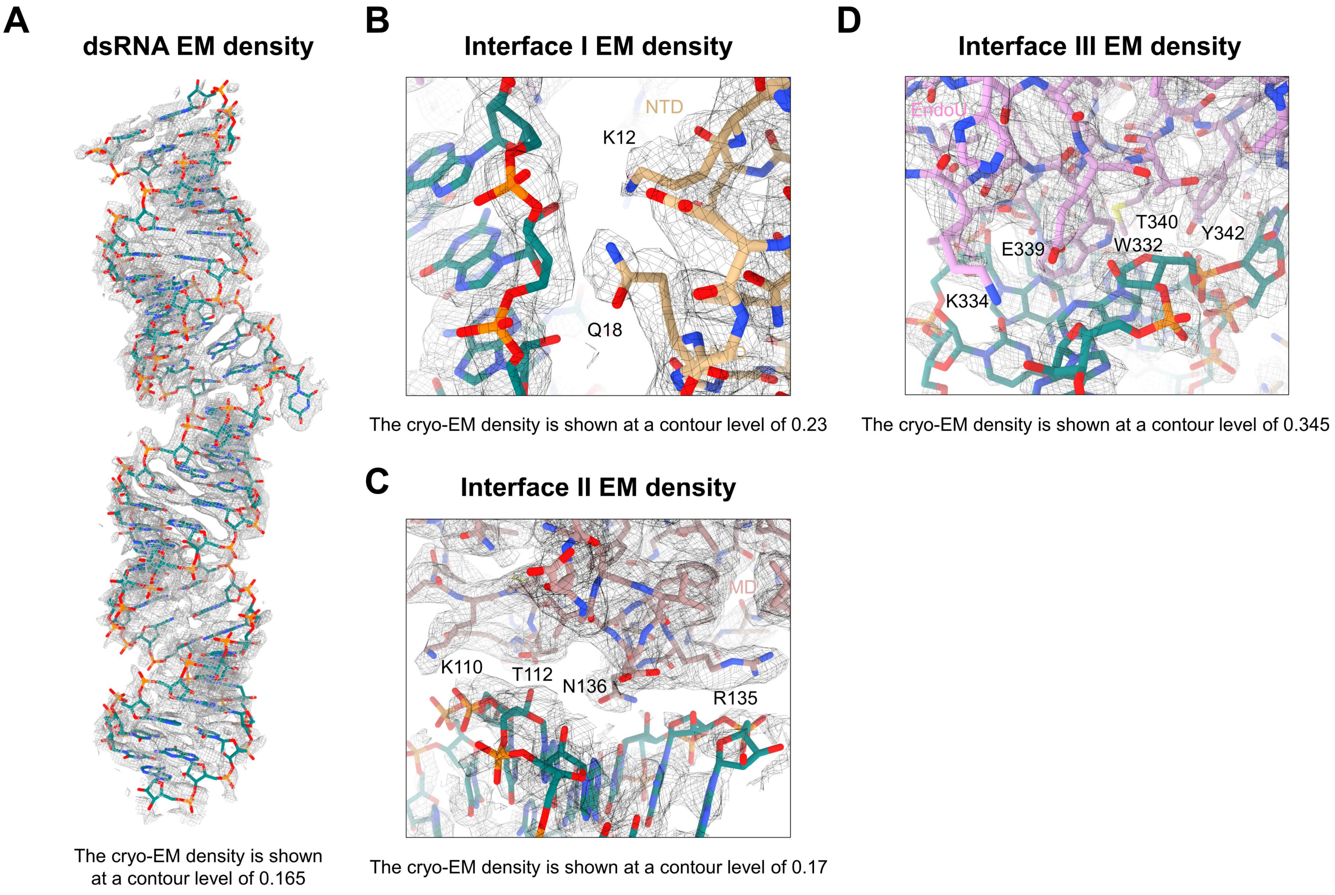

Supplementary Figure S8. Representative cryo-EM densities of nsp15/dsRNA complex. (**A**) Cryo-EM density of dsRNA present in the nsp15/dsRNA complex. (**B–D**) Cryo-EM densities of three RNA-nsp15 interface areas.

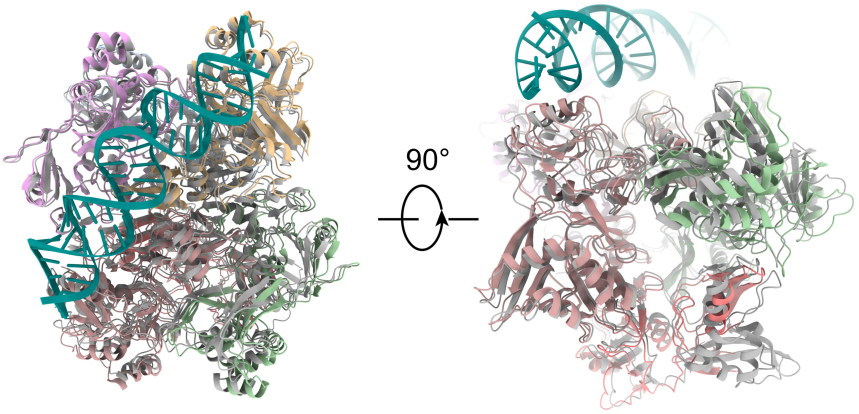

Supplementary Figure S9. Overlaid structures of the apo nsp15 (PDB 6VWW; gray) and our dsRNA-bound nsp15 complex (colored).

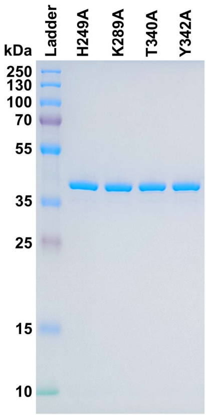

Supplementary Figure S10. SDS-PAGE analysis of purified recombinant SARS-CoV-2 nsp15 mutants.

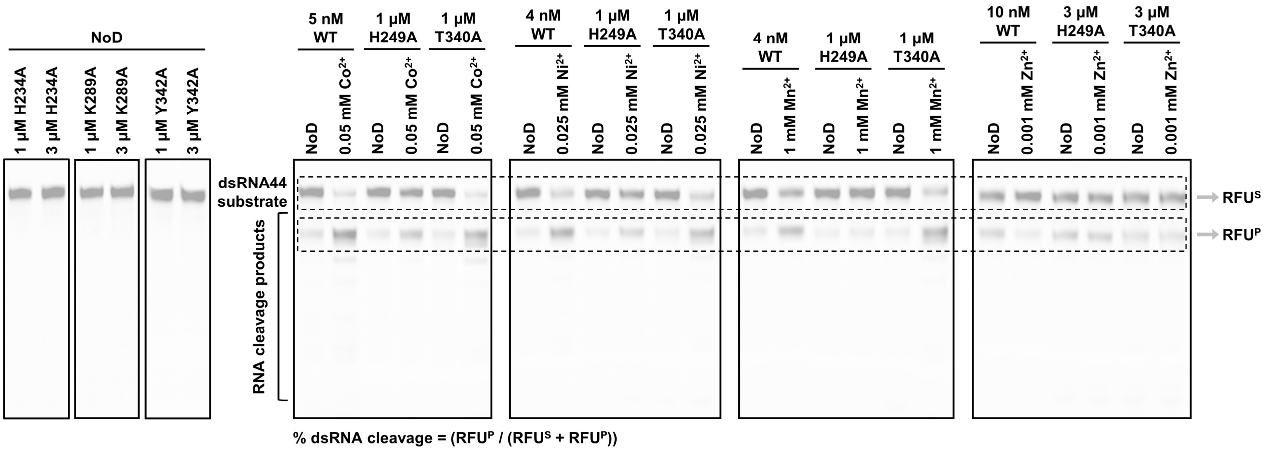

Supplementary Figure S11. Cleavage of the dsRNA44 substrate by wild-type (WT) SARS-CoV-2 nsp15 and its mutants under different conditions, related to Fig. 4E. NoD refers to no divalent cations. The quantification results are shown in Fig. 4E.

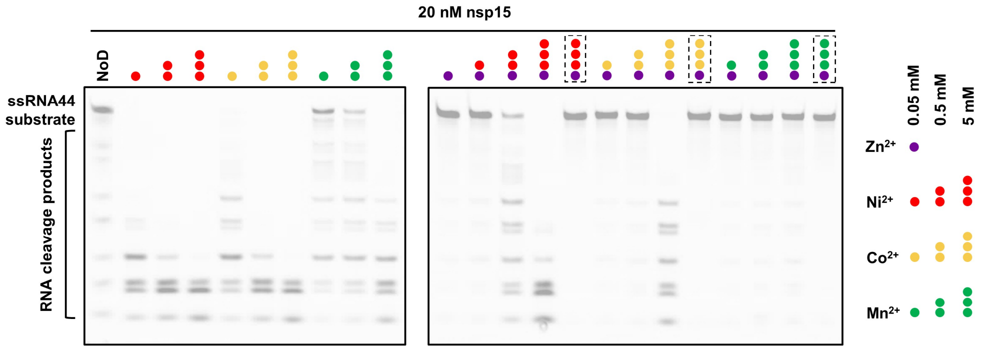

Supplementary Figure S12. Cleavage of the ssRNA44 substrate by SARS-CoV-2 nsp15 in the presence of various concentrations of Ni^{2+}, Co^{2+}, and Mn^{2+}, with or without 0.05 mM Zn^{2+}. NoD refers to no divalent cations. The reactions with the H234A mutant are marked with dashed boxes.

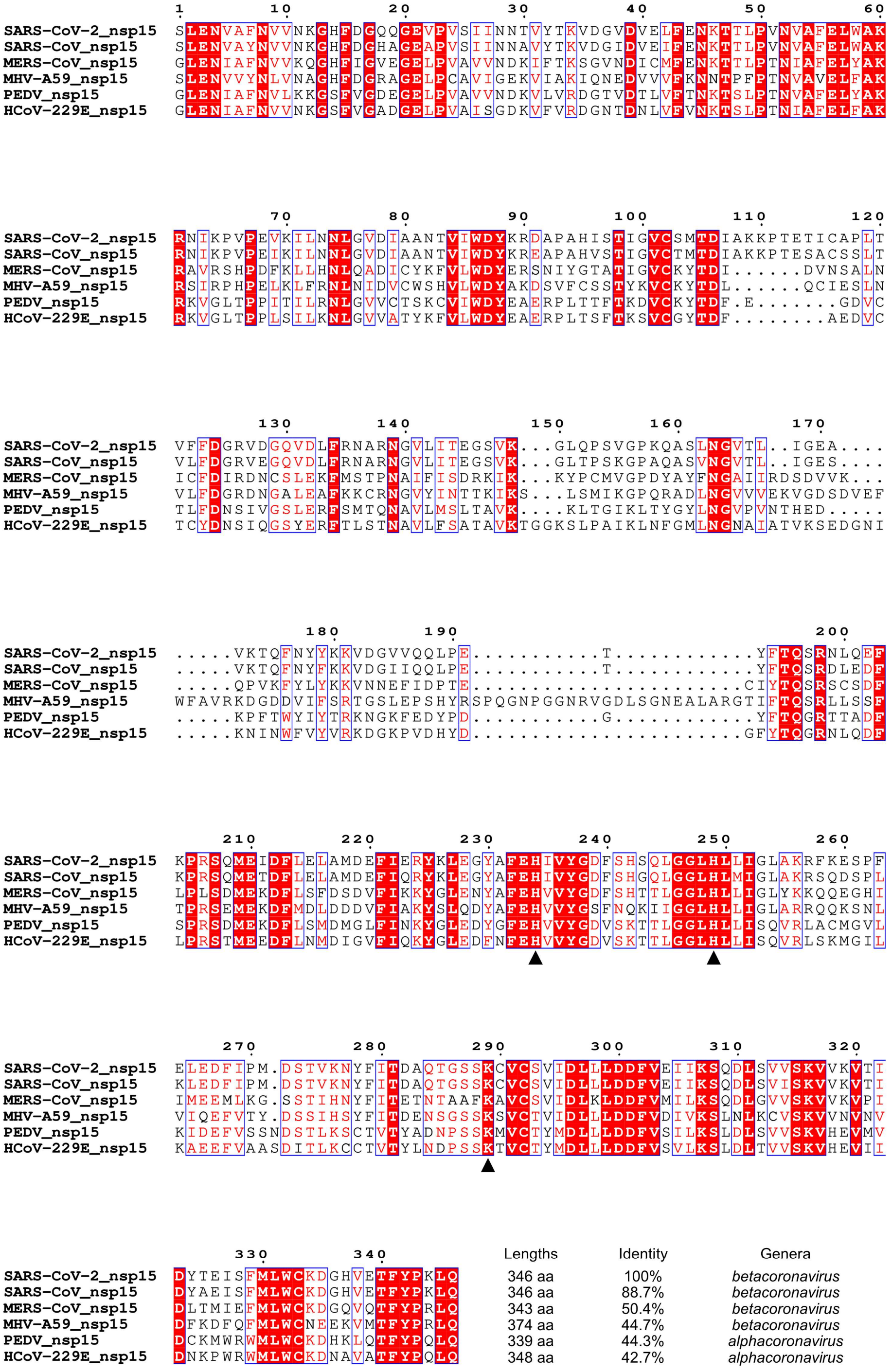

Supplementary Figure S13. Sequence alignment of nsp15 of various coronaviruses. The lengths and identity to SARS-CoV-2 nsp15 of these nsp15, as well as the genera of these coronaviruses are shown at the end of the sequences. The catalytic residues, including two histidine residues and one lysine residue, are marked with black triangles. The sequence alignment was performed using Geneious Basic 4.8.4 and ESPript 3.0 (https://espript.ibcp.fr/ESPript/cgi-bin/ESPript.cgi).

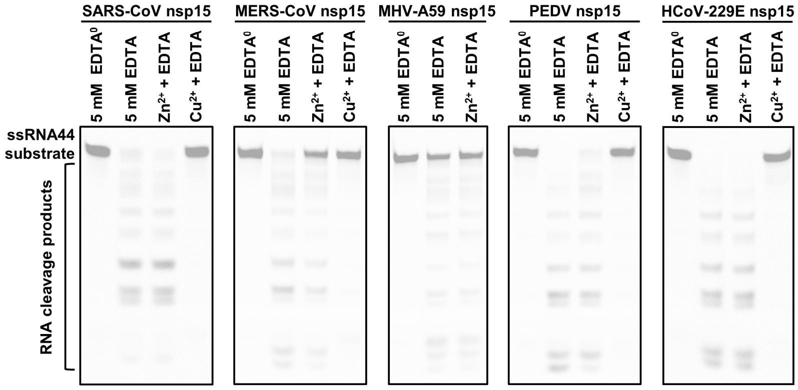

Supplementary Figure S14. Cleavage of the ssRNA44 substrate by nsp15 of various coronaviruses, with or without pretreatment of the nsp15 protein with 0.5 mM Zn^{2+} or Cu^{2+}. Zn^{2+} + EDTA and Cu^{2+} + EDTA refer to that 0.5 mM Zn^{2+} or Cu^{2+} was removed by the addition of 5 mM EDTA after incubation with the nsp15 protein and before substrate addition. The reactions lacking nsp15 are marked with ^{0}.

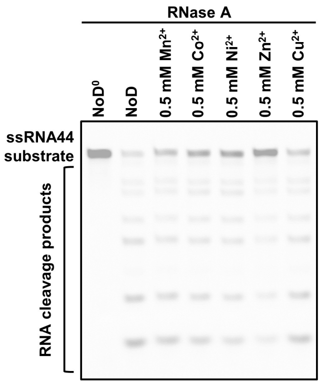

Supplementary Figure S15. Cleavage of the ssRNA44 substrate by RNase A in the absence of divalent cations (NoD) or in the presence of 0.5 mM Mn^{2+}, Co^{2+}, Ni^{2+}, Zn^{2+}, or Cu^{2+}. The reaction lacking RNase A is marked with ^{0}.

Supplementary Table S1. Complete sequence of the His-nsp15 construct (5' to 3').

TGGCGAATGGGACGCGCCCTGTAGCGGCGCATTAAGCGCGGCGGGTGTGGTGGTTACGCGCAGCGTGACCG<br>CTACACTTGCCAGCGCCCTAGCGCCCGCTCCTTTCGCTTTCTTCCCTTCCTTTCTCGCCACGTTCGCCGGCTTT<br>CCCCGTCAAGCTCTAAATCGGGGGCTCCCTTTAGGGTTCCGATTTAGTGCTTTACGGCACCTCGACCCCAAAA<br>AACTTGATTAGGGTGATGGTTCACGTAGTGGGCCATCGCCCTGATAGACGGTTTTTCGCCCTTTGACGTTGGA<br>GTCCACGTTCTTTAATAGTGGACTCTTGTTCCAAACTGGAACAACACTCAACCCTATCTCGGTCTATTCTTTTGA<br>TTTATAAGGGATTTTGCCGATTTCGGCCTATTGGTTAAAAAATGAGCTGATTTAACAAAAATTTAACGCGAATTTT<br>AACAAAATATTAACGTTTACAATTTCAGGTGGCACTTTTCGGGGAAATGTGCGCGGAACCCCTATTTGTTTATTT<br>TTCTAAATACATTCAAATATGTATCCGCTCATGAATTAATTCTTAGAAAAACTCATCGAGCATCAAATGAAACTGC<br>AATTTATTCATATCAGGATTATCAATACCATATTTTTGAAAAAGCCGTTTCTGTAATGAAGGAGAAAACTCACCG<br>AGGCAGTTCCATAGGATGGCAAGATCCTGGTATCGGTCTGCGATTCCGACTCGTCCAACATCAATACAACCTA<br>TTAATTTCCCCTCGTCAAAAATAAGGTTATCAAGTGAGAAATCACCATGAGTGACGACTGAATCCGGTGAGAAT<br>GGCAAAAGTTTATGCATTTCTTTCCAGACTTGTTCAACAGGCCAGCCATTACGCTCGTCATCAAAATCACTCGC<br>ATCAACCAAACCGTTATTCATTCGTGATTGCGCCTGAGCGAGACGAAATACGCGATCGCTGTTAAAAGGACAAT<br>TACAAACAGGAATCGAATGCAACCGGCGCAGGAACACTGCCAGCGCATCAACAATATTTTCACCTGAATCAGG<br>ATATTCTTCTAATACCTGGAATGCTGTTTTCCCGGGGATCGCAGTGGTGAGTAACCATGCATCATCAGGAGTAC<br>GGATAAAATGCTTGATGGTCGGAAGAGGCATAAATTCCGTCAGCCAGTTTAGTCTGACCATCTCATCTGTAACA<br>TCATTGGCAACGCTACCTTTGCCATGTTTCAGAAACAACTCTGGCGCATCGGGCTTCCCATACAATCGATAGAT<br>TGTCGCACCTGATTGCCCGACATTATCGCGAGCCCATTTATACCCATATAAATCAGCATCCATGTTGGAATTTA<br>ATCGCGGCCTAGAGCAAGACGTTTCCCGTTGAATATGGCTCATAACACCCCTTGTATTACTGTTTATGTAAGCA<br>GACAGTTTTATTGTTCATGACCAAAATCCCTTAACGTGAGTTTTCGTTCCACTGAGCGTCAGACCCCGTAGAAA<br>AGATCAAAGGATCTTCTTGAGATCCTTTTTTTCTGCGCGTAATCTGCTGCTTGCAAACAAAAAAACCACCGCTAC<br>CAGCGGTGGTTTGTTTGCCGGATCAAGAGCTACCAACTCTTTTTCCGAAGGTAACTGGCTTCAGCAGAGCGCA<br>GATACCAAATACTGTCCTTCTAGTGTAGCCGTAGTTAGGCCACCACTTCAAGAACTCTGTAGCACCGCCTACAT<br>ACCTCGCTCTGCTAATCCTGTTACCAGTGGCTGCTGCCAGTGGCGATAAGTCGTGTCTTACCGGGTTGGACTC<br>AAGACGATAGTTACCGGATAAGGCGCAGCGGTCGGGCTGAACGGGGGGTTCGTGCACACAGCCCAGCTTGG<br>AGCGAACGACCTACACCGAACTGAGATACCTACAGCGTGAGCTATGAGAAAGCGCCACGCTTCCCGAAGGGA<br>GAAAGGCGGACAGGTATCCGGTAAGCGGCAGGGTCGGAACAGGAGAGCGCACGAGGGAGCTTCCAGGGGG<br>AAACGCCTGGTATCTTTATAGTCCTGTCGGGTTTCGCCACCTCTGACTTGAGCGTCGATTTTTGTGATGCTCGT<br>CAGGGGGGCGGAGCCTATGGAAAAACGCCAGCAACGCGGCCTTTTTACGGTTCCTGGCCTTTTGCTGGCCTT<br>TTGCTCACATGTTCTTTCCTGCGTTATCCCCTGATTCTGTGGATAACCGTATTACCGCCTTTGAGTGAGCTGATA<br>CCGCTCGCCGCAGCCGAACGACCGAGCGCAGCGAGTCAGTGAGCGAGGAAGCGGAAGAGCGCCTGATGCG<br>GTATTTTCTCCTTACGCATCTGTGCGGTATTTCACACCGCATATATGGTGCACTCTCAGTACAATCTGCTCTGAT<br>GCCGCATAGTTAAGCCAGTATACACTCCGCTATCGCTACGTGACTGGGTCATGGCTGCGCCCCGACACCCGC<br>CAACACCCGCTGACGCGCCCTGACGGGCTTGTCTGCTCCCGGCATCCGCTTACAGACAAGCTGTGACCGTCT<br>CCGGGAGCTGCATGTGTCAGAGGTTTTCACCGTCATCACCGAAACGCGCGAGGCAGCTGCGGTAAAGCTCAT<br>CAGCGTGGTCGTGAAGCGATTCACAGATGTCTGCCTGTTCATCCGCGTCCAGCTCGTTGAGTTTCTCCAGAAG<br>CGTTAATGTCTGGCTTCTGATAAAGCGGGCCATGTTAAGGGCGGTTTTTTCCTGTTTGGTCACTGATGCCTCCG<br>TGTAAGGGGGATTTCTGTTCATGGGGGTAATGATACCGATGAAACGAGAGAGGATGCTCACGATACGGGTTAC<br>TGATGATGAACATGCCCGGTTACTGGAACGTTGTGAGGGTAAACAACTGGCGGTATGGATGCGGCGGGACCA<br>GAGAAAAATCACTCAGGGTCAATGCCAGCGCTTCGTTAATACAGATGTAGGTGTTCCACAGGGTAGCCAGCAG<br>CATCCTGCGATGCAGATCCGGAACATAATGGTGCAGGGCGCTGACTTCCGCGTTTCCAGACTTTACGAAACAC<br>GGAAACCGAAGACCATTCATGTTGTTGCTCAGGTCGCAGACGTTTTGCAGCAGCAGTCGCTTCACGTTCGCTC<br>GCGTATCGGTGATTCATTCTGCTAACCAGTAAGGCAACCCCGCCAGCCTAGCCGGGTCCTCAACGACAGGAG<br>CACGATCATGCGCACCCGTGGGGCCGCCATGCCGGCGATAATGGCCTGCTTCTCGCCGAAACGTTTGGTGGC<br>GGGACCAGTGACGAAGGCTTGAGCGAGGGCGTGCAAGATTCCGAATACCGCAAGCGACAGGCCGATCATCG<br>TCGCGCTCCAGCGAAAGCGGTCCTCGCCGAAAATGACCCAGAGCGCTGCCGGCACCTGTCCTACGAGTTGCA<br>TGATAAAGAAGACAGTCATAAGTGCGGCGACGATAGTCATGCCCCGCGCCCACCGGAAGGAGCTGACTGGGT<br>TGAAGGCTCTCAAGGGCATCGGTCGAGATCCCGGTGCCTAATGAGTGAGCTAACTTACATTAATTGCGTTGCG

CTCACTGCCCGCTTTCCAGTCGGGAAACCTGTCGTGCCAGCTGCATTAATGAATCGGCCAACGCGCGGGGAG
AGGCGGTTTGCGTATTGGGCGCCAGGGTGGTTTTTCTTTTCACCAGTGAGACGGGCAACAGCTGATTGCCCTT
CACCGCCTGGCCCTGAGAGAGTTGCAGCAAGCGGTCCACGCTGGTTTGCCCCAGCAGGCGAAAATCCTGTTT
GATGGTGGTTAACGGCGGGATATAACATGAGCTGTCTTCGGTATCGTCGTATCCCACTACCGAGATATCCGCA
CCAACGCGCAGCCCGGACTCGGTAATGGCGCGCATTGCGCCCAGCGCCATCTGATCGTTGGCAACCAGCATC
GCAGTGGGAACGATGCCCTCATTCAGCATTTGCATGGTTTGTTGAAAACCGGACATGGCACTCCAGTCGCCTT
CCCGTTCCGCTATCGGCTGAATTTGATTGCGAGTGAGATATTTATGCCAGCCAGCCAGACGCAGACGCGCCGA
GACAGAACTTAATGGGCCCGCTAACAGCGCGATTTGCTGGTGACCCAATGCGACCAGATGCTCCACGCCCAG
TCGCGTACCGTCTTCATGGGAGAAAATAATACTGTTGATGGGTGTCTGGTCAGAGACATCAAGAAATAACGCC
GGAACATTAGTGCAGGCAGCTTCCACAGCAATGGCATCCTGGTCATCCAGCGGATAGTTAATGATCAGCCCAC
TGACGCGTTGCGCGAGAAGATTGTGCACCGCCGCTTTACAGGCTTCGACGCCGCTTCGTTCTACCATCGACAC
CACCACGCTGGCACCCAGTTGATCGGCGCGAGATTTAATCGCCGCGACAATTTGCGACGGCGCGTGCAGGGC
CAGACTGGAGGTGGCAACGCCAATCAGCAACGACTGTTTGCCCGCCAGTTGTTGTGCCACGCGGTTGGGAAT
GTAATTCAGCTCCGCCATCGCCGCTTCCACTTTTTCCCGCGTTTTCGCAGAAACGTGGCTGGCCTGGTTCACC
ACGCGGGAAACGGTCTGATAAGAGACACCGGCATACTCTGCGACATCGTATAACGTTACTGGTTTCACATTCA
CCACCCTGAATTGACTCTCTTCCGGGCGCTATCATGCCATACCGCGAAAGGTTTTGCGCCATTCGATGGTGTC
CGGGATCTCGACGCTCTCCCTTATGCGACTCCTGCATTAGGAAGCAGCCCAGTAGTAGGTTGAGGCCGTTGA
GCACCGCCGCCGCAAGGAATGGTGCATGCAAGGAGATGGCGCCCAACAGTCCCCCGGCCACGGGGCCTGCC
ACCATACCCACGCCGAAACAAGCGCTCATGAGCCCGAAGTGGCGAGCCCGATCTTCCCCATCGGTGATGTCG
GCGATATAGGCGCCAGCAACCGCACCTGTGGCGCCGGTGATGCCGGCCACGATGCGTCCGGCGTAGAGGAT
CGAGATCTCGATCCCGCGAAATTAATACGACTCACTATAGGGGAATTGTGAGCGGATAACAATTCCCCTCTAGA
AATAATTTTGTTTAACTTTAAGAAGGAGATATACCATGGGCAGCAGCCATCATCATCATCATCACAGCAGCGGC
AGCCTGGAAAACGTTGCGTTTAACGTGGTTAACAAAGGTCACTTCGATGGTCAGCAAGGCGAGGTGCCGGTTA
GCATCATTAACAACACCGTGTACACCAAGGTTGACGGCGTGGATGTTGAGCTGTTTGAAAACAAAACCACCCT
GCCGGTGAACGTTGCGTTCGAGCTGTGGGCGAAGCGTAACATCAAACCGGTGCCGGAAGTTAAGATTCTGAA
CAACCTGGGTGTGGACATCGCGGCGAACACCGTTATTTGGGACTATAAACGTGATGCGCCGGCGCACATCAG
CACCATTGGCGTTTGCAGCATGACCGATATCGCGAAGAAACCGACCGAAACCATTTGCGCGCCGCTGACCGT
GTTCTTTGACGGTCGTGTGGATGGCCAGGTTGACCTGTTTCGTAACGCGCGTAACGGTGTGCTGATCACCGAG
GGTAGCGTTAAAGGTCTGCAGCCGAGCGTGGGTCCGAAACAAGCGAGCCTGAACGGTGTTACCCTGATTGGC
GAAGCGGTGAAGACCCAGTTCAACTACTATAAGAAAGTTGACGGTGTGGTTCAGCAACTGCCGGAGACCTACT
TTACCCAGAGCCGTAACCTGCAAGAATTCAAGCCGCGTAGCCAAATGGAGATCGATTTTCTGGAACTGGCGAT
GGACGAGTTCATTGAACGTTACAAACTGGAGGGTTATGCGTTTGAACACATCGTTTACGGCGATTTCAGCCATA
GCCAGCTGGGTGGCCTGCACCTGCTGATTGGTCTGGCGAAGCGTTTCAAAGAGAGCCCGTTTGAGCTGGAAG
ATTTCATCCCGATGGACAGCACCGTGAAGAACTATTTTATTACCGATGCGCAGACCGGCAGCAGCAAATGCGT
GTGCAGCGTTATCGACCTGCTGCTGGACGATTTCGTTGAAATCATTAAAAGCCAAGATCTGAGCGTGGTTAGC
AAGGTGGTTAAAGTGACCATCGATTACACCGAGATTAGCTTTATGCTGTGGTGCAAGGACGGCCACGTGGAAA
CCTTCTATCCGAAACTGCAATAAGAATTCGAGCTCCGTCGACAAGCTTGCGGCCGCACTCGAGCACCACCACC
ACCACCACTGAGATCCGGCTGCTAACAAAGCCCGAAAGGAAGCTGAGTTGGCTGCTGCCACCGCTGAGCAAT
AACTAGCATAACCCCTTGGGGCCTCTAAACGGGTCTTGAGGGGTTTTTTGCTGAAAGGAGGAACTATATCCGG
AT

Supplementary Table S2. Primers for cloning.

Primers	Primer sequences (5’ to 3’)
F-Strep-tag II	TGGAGCCACCCGCAGTTCGAAAAGAGCAGCGGCAGCCTGG
R-Strep-tag II	CTTTTCGAACTGCGGGTGGCTCCAGCTGCTGCCCATGGTATATCTCC
F-H234A	TTTGAAGCGATCGTTTACGGCGATTTCAGCCATAG
R-H234A	AACGATCGCTTCAAACGCATAACCCTCCAGTTTGT
F-H249A	GGCCTGGCGCTGCTGATTGGTCTGGC
R-H249A	CAGCAGCGCCAGGCCACCCAGCTGGC
F-K289A	AGCAGCGCGTGCGTGTGCAGCGTTATCGAC
R-K289A	CACGCACGCGCTGCTGCCGGTCTGC
F-T340A	GTGGAAGCGTTCTATCCGAAACTGCAATAAGAATTCGAGC
R-T340A	ATAGAACGCTTCCACGTGGCCGTCCTTGC
F-Y342A	ACCTTCGCGCCGAAACTGCAATAAGAATTCGAGC
R-Y342A	TTTCGGCGCGAAGGTTTCCACGTGGCCG

Supplementary Table S3. Cryo-EM data collection, refinement and validation statistics.

	6:1 nsp15/dsRNA	6:2 nsp15/dsRNA
EMDB ID	63618	63619
PDB ID	9M48	9M49
**Data collection and processing**		
Magnification	105,000	
Voltage (kV)	300	
Electron exposure (e–/Å^2)	50	
Defocus range (μm)	-1.5 to -1.8	
Pixel size (Å)	0.84	
Symmetry imposed	C1	C1
Initial particle images (no.)	319,161	319,161
Final particle images (no.)	244,604	117,130
Map resolution (Å)	2.6	2.78
FSC threshold	0.143	0.143
Map resolution range (Å)	2.9 to 6.1	3.2 to 6.7
**Refinement**		
Initial model used (PDB)	7TJ2	9M48
Model resolution (Å)	2.8	3.2
FSC threshold	0.5	0.5
Model resolution range (Å)	50-2.8	50-3.2
Map sharpening *B* factor (Å^2)	-67.2	-67.2
Model composition		
Non-hydrogen atoms	16977	18285
Protein residues	1989	1989
Nucleotide residues	62	124
Ligands	0	0
B factors (Å^2)		
Protein	54.65	70.26
Nucleotide	21.48	27.67
Ligand	0	0
R.m.s. deviations		
Bond lengths (Å)	0.002	0.002
Bond angles (°)	0.407	0.456
Validation		
MolProbity score	1.38	1.36
Clashscore	4.5	5.41
Poor rotamers (%)	1.08	1.25
Ramachandran plot		
Favored (%)	97.31	98.07
Allowed (%)	2.69	1.93
Disallowed (%)	0	0
